# A Clinically Accessible Nomogram for Identifying Likely Psoriatic Arthritis Among Patients with Psoriasis: A Multicenter Cross-Sectional Study

**DOI:** 10.3390/jcm15166215

**Published:** 2026-08-11

**Authors:** Qing Guo, Hong Luan, Xiaohong Chen, Xi Zhao, Mouhsun Chiang, Ling Wu, Xixi Tan, Xue Min, Xiaolin Feng, Bo Xiao, Mi Chen, Jialin Lin, Zhenying Zhang

**Affiliations:** 1 Department of Dermatology, The University of Hong Kong-Shenzhen Hospital, Shenzhen 518053, China; guoq@hku-szh.org (Q.G.); luanh8@hku-szh.org (H.L.); chenxh9@hku-szh.org (X.C.); tanxx@hku-szh.org (X.T.); minx@hku-szh.org (X.M.); linjl@hku-szh.org (J.L.); 2 Department of Rheumatology, The University of Hong Kong-Shenzhen Hospital, Shenzhen 518053, China; zhaox@hku-szh.org (X.Z.); wul@hku-szh.org (L.W.); fengxl@hku-szh.org (X.F.); amyxiaobo@163.com (B.X.); chenmi@hku-szh.org (M.C.); 3 Department of Ultrasound, The University of Hong Kong-Shenzhen Hospital, Shenzhen 518053, China; jiangmx@hku-szh.org

**Keywords:** psoriatic arthritis, psoriasis, nomogram, screening tool, clinical decision support

## Abstract

**Background/Objectives:** Psoriatic arthritis (PsA) affects up to 30% of psoriasis patients but remains underdiagnosed due to heterogeneous early manifestations. Existing tools rely on advanced imaging or biomarkers, limiting feasibility. This study aimed to develop a simple nomogram, using routinely available clinical features to identify patients with likely PsA. **Methods:** This multicenter cross-sectional study enrolled adult psoriasis patients from two tertiary hospitals (training: *n* = 884; validation: *n* = 690). PsA was diagnosed using the Classification Criteria for Psoriatic Arthritis (CASPAR) by rheumatologists and dermatologists who were blinded to each other’s assessments. Thirteen candidate predictors, including demographics, disease history, and lesion characteristics, were prespecified. Independent predictors were selected using least absolute shrinkage and selection operator (LASSO) regression, followed by multivariable logistic regression. Internal validation used 1000 bootstrap resamples, followed by temporal–geographic validation. Performance was assessed using area under the curve (AUC), calibration plots, the Hosmer–Lemeshow test, decision curve analysis (DCA), and clinical impact curves (CIC). Sensitivity analyses included E-value analysis for unmeasured confounding and alternative model specifications. **Results:** Five independent predictors were identified: uveitis (odds ratio (OR): 5.23, 95% confidence interval (CI): 1.94–14.10), inverse lesions (OR: 1.65, 95% CI: 1.09–2.48), scalp lesions (OR: 2.97, 95% CI: 1.45–6.10), nail lesions (OR: 5.98, 95% CI: 3.69–9.68), and arthralgia (OR: 20.23, 95% CI: 13.14–31.13). The nomogram showed good discrimination (AUC: 0.885 training, 0.874 validation), with good calibration and clinical utility confirmed by DCA and CIC. **Conclusions:** This nomogram, integrating five clinically accessible predictors, offers a practical tool to identify psoriasis patients with likely PsA, supporting timely referral in tertiary dermatology outpatient settings.

## 1. Introduction

Psoriatic arthritis (PsA), a chronic inflammatory arthritis linked to psoriasis, affects approximately 30% of psoriasis patients and manifests as joint inflammation, enthesitis, and dactylitis [1]. Without timely intervention, PsA can lead to irreversible joint damage, functional disability, and increased risk of cardiovascular disease, metabolic syndrome, and depression [2,3,4]. Early diagnosis and intervention are critical to mitigating these outcomes [5]. However, the disease’s multifactorial pathogenesis driven by genetic susceptibility, immune dysregulation, and environmental triggers complicates early recognition [6]. Heterogeneous clinical presentations and the absence of specific biomarkers further obscure diagnosis, often resulting in delays or misclassification, particularly in settings where specialist resources are limited [7].

Recognized clinical features associated with PsA among psoriasis patients include cutaneous and extracutaneous manifestations. Scalp lesions, nail lesions, and inverse lesions are strongly linked to the presence of PsA [8,9]. Extensive skin involvement, uveitis, and familial psoriasis history are also more prevalent in patients with PsA [10]. Additionally, modifiable factors such as smoking and obesity have been associated with increased PsA risk [11,12]. However, as of yet, no single clinical feature can reliably distinguish psoriasis patients with PsA from those without it.

To facilitate early identification, several screening questionnaires have been developed, including the PsA Screening and Evaluation (PASE) questionnaire [13] and the Toronto PsA Screen (ToPAS) [14]. These tools are relatively simple to apply and can help identify patients who warrant further evaluation. Nevertheless, they rely on patient-reported symptoms, which may be subject to recall bias, and their performance varies across populations [15]. Moreover, they do not provide a quantitative, individualized probability estimate.

Current diagnostic criteria, such as the Classification Criteria for Psoriatic Arthritis (CASPAR), can only be applied after a clinician has confirmed the presence of inflammatory musculoskeletal disease; thus, they are not suitable for identifying psoriasis patients who may already have early or subclinical PsA but lack definitive arthritis signs [16]. Moreover, existing models for PsA identification face limitations, including self-reported questionnaires prone to misinterpretation and recall bias [15], small sample sizes [13], inadequate temporal–geographic validation [17], and reliance on inaccessible biomarkers [18], limiting their feasibility.

Nomograms offer a promising alternative by integrating multiple predictors into a visual, point-based tool that provides individualized probability estimates. However, existing nomograms for PsA still face similar barriers. Therefore, we developed and validated a practical nomogram using only routinely available clinical features to help clinicians identify psoriasis patients with likely PsA who would benefit from timely referral.

## 2. Methods

This multicenter cross-sectional study was approved by the institutional review boards of all participating centers and conducted in accordance with the ethical principles of the Declaration of Helsinki. Reporting adhered to the Transparent Reporting of a Multivariable Prediction Model for Individual Prognosis or Diagnosis (TRIPOD) statement [19,20,21] (see Checklist in File S1).

### 2.1. Patients

Patients were recruited from two urban tertiary hospitals in Shenzhen, China. Center 1 (the University of Hong Kong-Shenzhen Hospital) is a 2000-bed hospital that primarily serves a mix of local residents and expatriates. Center 2 (the Eighth Affiliated Hospital of Sun Yat-sen University) is a 1500-bed hospital that predominantly serves the local urban population. The training/internal validation cohort was enrolled at Center 1 between December 2021 and December 2023. The temporal–geographic cohort was recruited at Center 2 between December 2022 and December 2024. All patients were independently diagnosed by rheumatologists and dermatologists who were blinded to each other’s assessments using the CASPAR criteria [16]. Before applying the CASPAR criteria, the presence of inflammatory musculoskeletal disease was assessed by rheumatologists through a standardized clinical evaluation, including peripheral joint symptoms and signs, focused joint examination, assessment of dactylitis and enthesitis when clinically indicated, and evaluation of axial symptoms when present. When diagnostic uncertainty remained, ultrasound examination was performed by a dedicated musculoskeletal ultrasound specialist (M.C.) to support clinical judgment. Standardized training was provided to all research staff to ensure consistent data collection across sites.

### 2.2. Inclusion and Exclusion Criteria

Inclusion criteria: (1) age ≥18 years; (2) any type of psoriasis; (3) provision of written informed consent and willingness to comply with study procedures.

Exclusion criteria: (1) concomitant rheumatic or arthritic conditions (e.g., rheumatoid arthritis, reactive arthritis, inflammatory bowel disease-associated arthritis, axial spondyloarthritis, gout, calcium pyrophosphate deposition disease, or osteoarthritis); (2) incomplete medical records; (3) >10% missing data for key variables; and (4) pregnancy or lactation.

All patients had at least one dermatology outpatient visit with a hospital information system (HIS) diagnosis of psoriasis.

### 2.3. Data Collection

Thirteen candidate predictors were prespecified based on clinical relevance and routine availability: demographics (sex, age, educational level, body mass index (BMI), smoking history) and clinical features (psoriasis duration, family history of psoriasis, uveitis, arthralgia, body surface area (BSA), scalp lesions, nail lesions, inverse lesions). Nail lesions were defined as pitting, onycholysis, or subungual hyperkeratosis. Inverse lesions were defined as psoriasis involving intertriginous regions (e.g., axillae, groin). Uveitis was based on ophthalmologist diagnosis. Arthralgia was identified by physician assessment based on patient-reported joint pain in the preceding 4 weeks, combined with a focused musculoskeletal physical examination. All predictors were extracted from the earliest dermatology visit within the enrollment window and coded as follows: binary (0/1) for sex, smoking history, family history of psoriasis, uveitis, arthralgia, scalp lesions, nail lesions, and inverse lesions; continuous for age, psoriasis duration, BMI, and BSA; and ordinal (1–3) for educational level.

### 2.4. Sample Size Calculation

Sample size was determined based on established guidance for model development and validation [22,23,24,25,26]. For the training set, assuming a target C-statistic of 0.75 (reflecting the lower bound of acceptable discrimination for clinical prediction models [27]) and 90% shrinkage to account for potential overfitting in multivariable logistic regression [22], a minimum of 866 participants was required. For temporal–geographic, considering desired precision for discrimination, calibration, and overall performance estimates, a minimum of 688 participants was needed. The final sample of 1574 patients exceeded both requirements. Full details of the calculation procedure and R code are provided in File S2 (see Supporting Information).

### 2.5. Statistical Analysis

All analyses were performed using R software (version 4.4.2; https://www.r-project.org/), with a two-sided *p* < 0.05 considered statistically significant. Detailed information on R packages is provided in File S3 (see Supporting Information).

Missing data (<10% per variable) were addressed using multiple imputation by chained equations (*MICE*) in the mice package (version 3.16.0) in R (5 imputations) to minimize bias [28]. Normality of continuous variables was assessed using the Kolmogorov–Smirnov test. Normally distributed variables were summarized as mean ± standard deviation (SD) and non-normally distributed variables as median (range). Categorical variables were reported as frequencies (%). Group comparisons used Student’s *t*-test or Mann–Whitney U test for continuous variables, and χ^2^ or Fisher’s exact test for categorical variables, as appropriate.

Variable selection was performed using least absolute shrinkage and selection operator (LASSO) regression with 10-fold cross-validation, with optimal λ values determined by the minimum criteria and the 1 standard error criteria. Variables with non-zero coefficients at the more stringent *lambda.1se* were entered into multivariable logistic regression, and independent predictors were incorporated into the nomogram.

Model performance was evaluated across three domains. Discrimination was assessed using the area under the receiver operating characteristic curve (AUC), with an AUC > 0.7 considered acceptable. Calibration was examined through calibration plots and the Hosmer–Lemeshow goodness-of-fit test, where a *p* > 0.05 indicates adequate agreement between predicted and observed outcomes [29]. Clinical utility was evaluated using decision curve analysis (DCA) and clinical impact curves (CIC). Internal validation was performed using 1000 bootstrap resamples to correct for overfitting [30]. Temporal–geographic validation was conducted in the independent cohort from Center 2 to assess model performance.

To assess the robustness of our findings against potential unmeasured confounding, we performed an E-value analysis [31]. Additionally, pre-specified sensitivity analyses were conducted to evaluate the stability of the model under alternative specifications.

## 3. Results

### 3.1. Baseline Characteristics

The study population flow diagram is shown in Figure S1 (see Supporting Information). The training/internal validation cohort included 884 patients with psoriasis, of whom 221 (25.0%) met the CASPAR criteria for PsA at the study assessment. The temporal–geographic cohort comprised 690 psoriasis patients, with 163 (23.6%) diagnosed with PsA at the study assessment. Demographic and clinical characteristics of both cohorts are summarized in Table 1.

### 3.2. Development of the PsA Identification Model

**Figure 1:**
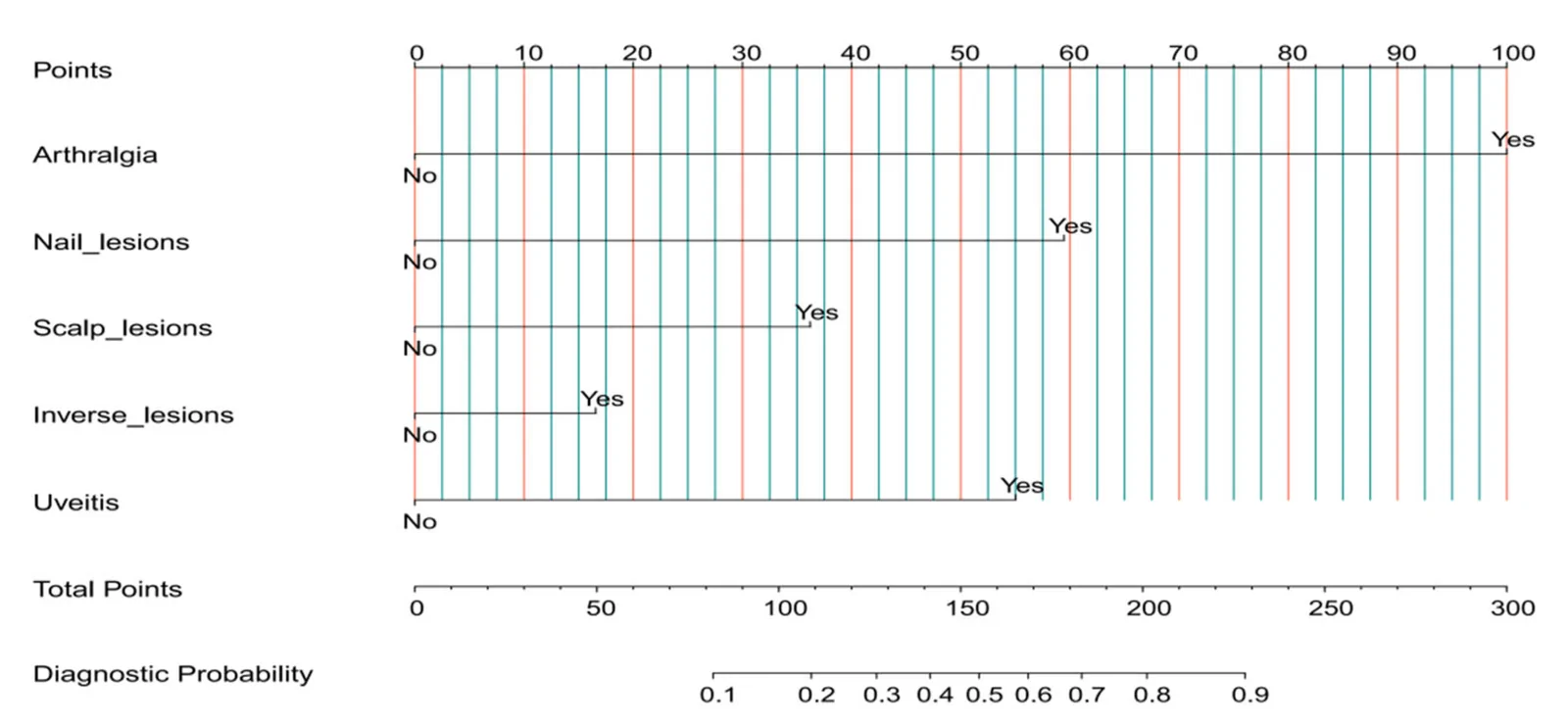
**Nomogram for estimating the probability of psoriatic arthritis (PsA) in patients with psoriasis.** The nomogram was developed based on five independent clinical predictors: Uveitis, inverse lesions, scalp lesions, nail lesions, and arthralgia. To use the nomogram, locate the point value corresponding to each patient’s clinical feature on the upper scale, sum the points, and draw a line down to the bottom scale to obtain the estimated probability of having PsA.

LASSO regression selected five predictors: inverse lesions, scalp lesions, nail lesions, arthralgia, and uveitis (Figure S2, see Supporting Information). Multivariable logistic regression confirmed that all five predictors were independently associated with PsA (Table 2). The logistic regression model is expressed as: logit(PsA) = −4.662 + 1.655 × uveitis + 0.498 × inverse lesions + 1.089 × scalp lesions + 1.788 × nail lesions + 3.007 × arthralgia. All variance inflation factors (VIFs) ranged from 1.01 to 1.07, confirming the absence of multicollinearity. These variables were integrated into a weighted nomogram (Figure 1) to support the clinical identification of psoriasis patients who are likely to have PsA.

### 3.3. Performance of the PsA Identification Nomogram

#### 3.3.1. Discrimination

**Figure 2:**
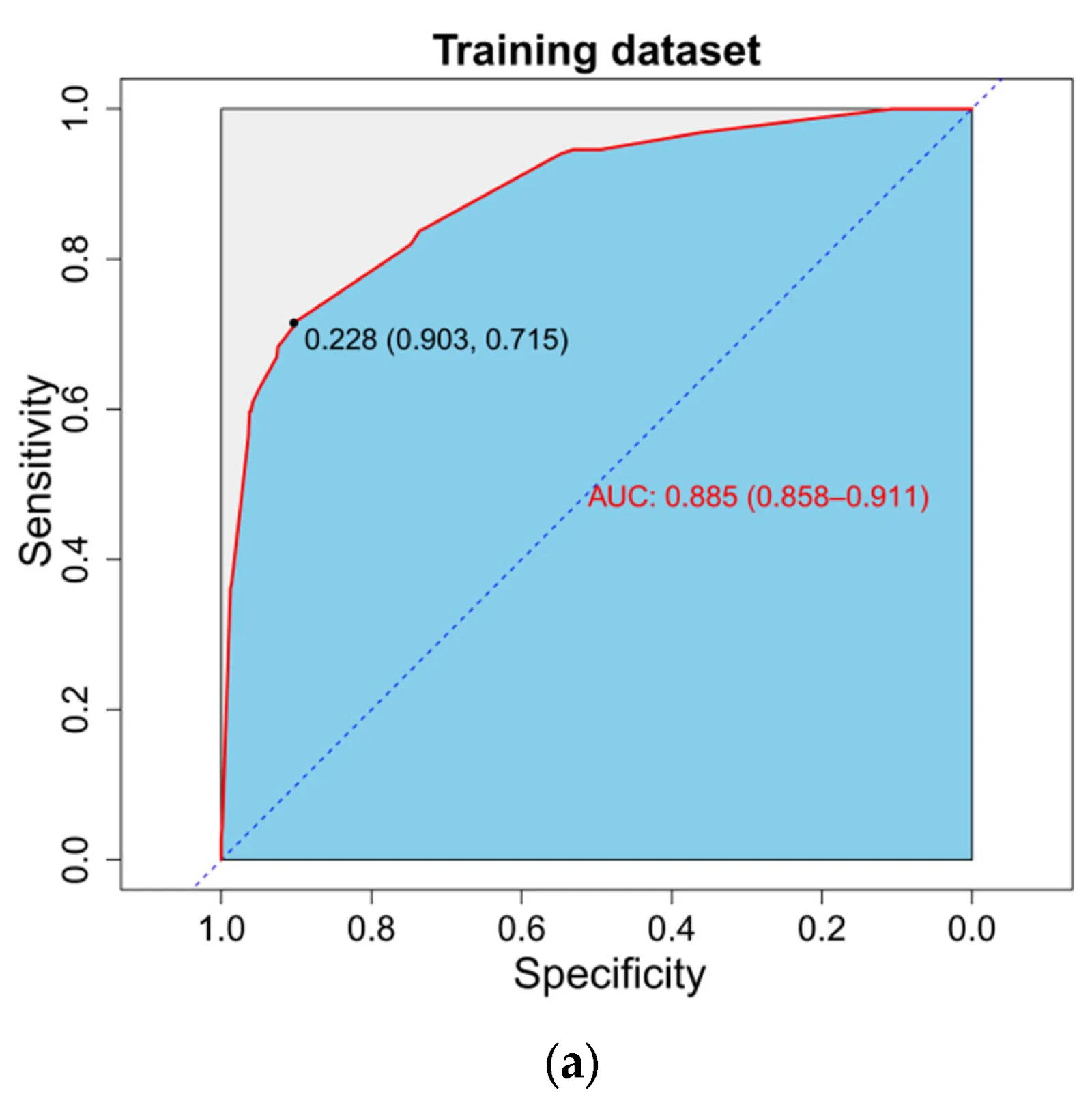

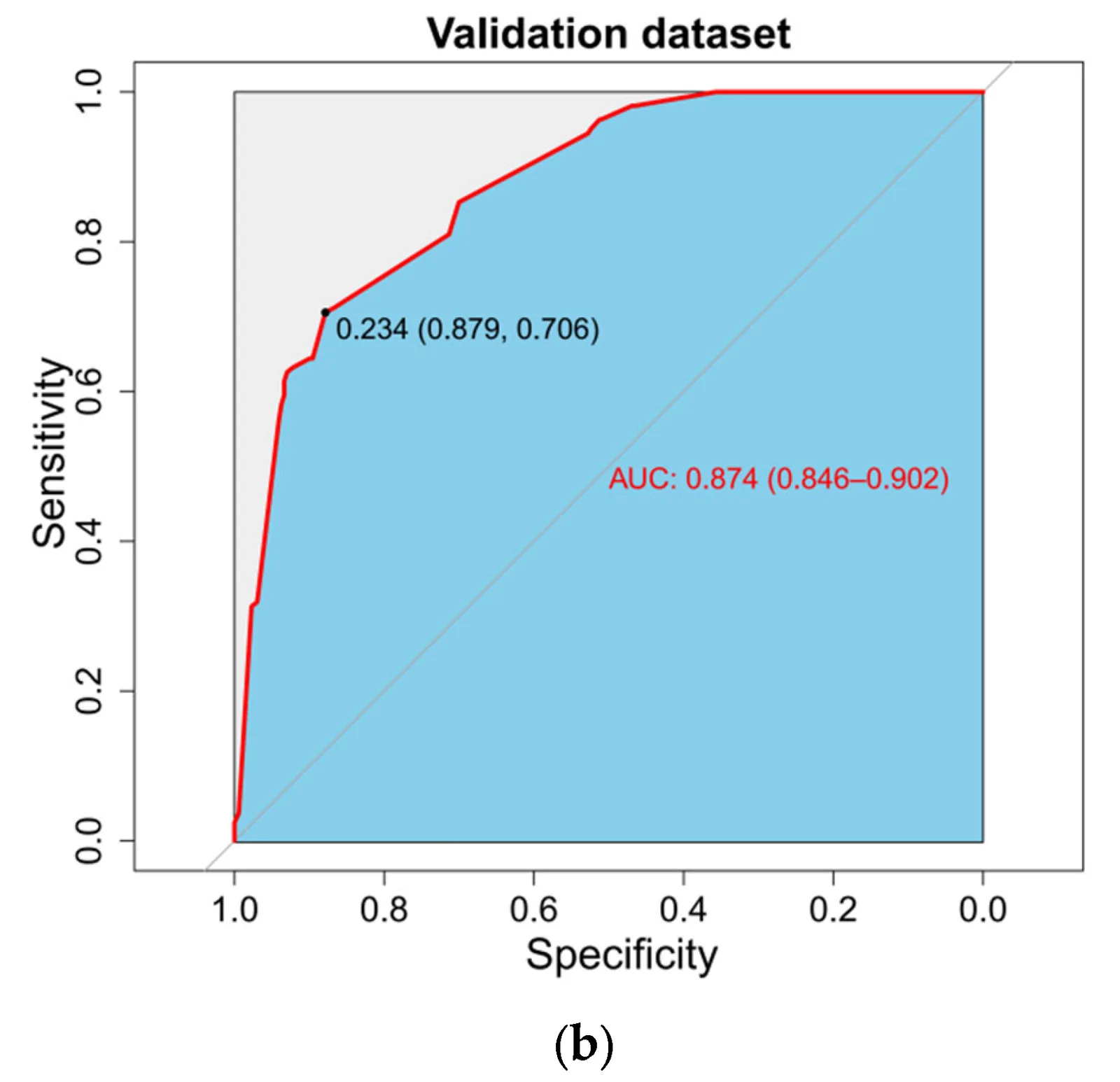
**ROC analysis of the clinical nomogram in training and validation cohorts.** (**a**) ROC curves in training dataset. (**b**) ROC curves in validation dataset.

The nomogram demonstrated good discrimination, with an AUC of 0.885 (95% CI 0.858–0.911) in the training cohort (Figure 2a) and 0.874 (95% CI 0.846–0.902) in the temporal–geographic cohort (Figure 2b). Optimal probability thresholds, determined using the Youden index, were 0.228 for the training cohort (sensitivity 71.5%, specificity 90.3%) and 0.234 for the validation cohort (sensitivity 70.6%, specificity 87.9%).

Internal validation using 1000 bootstrap resamples yielded a calibrated AUC of 0.883 (95% CI 0.856–0.909) (Figure S3, see Supporting Information), confirming model stability. In subgroup analyses by sex and age, the nomogram showed consistent discrimination, with AUCs ranging from 0.852 to 0.911. Confusion matrices further validated the nomogram’s accuracy, with 86.9% of the cases correctly classified in the training cohort and 85.4% in the validation cohort.

#### 3.3.2. Calibration

**Figure 3:**
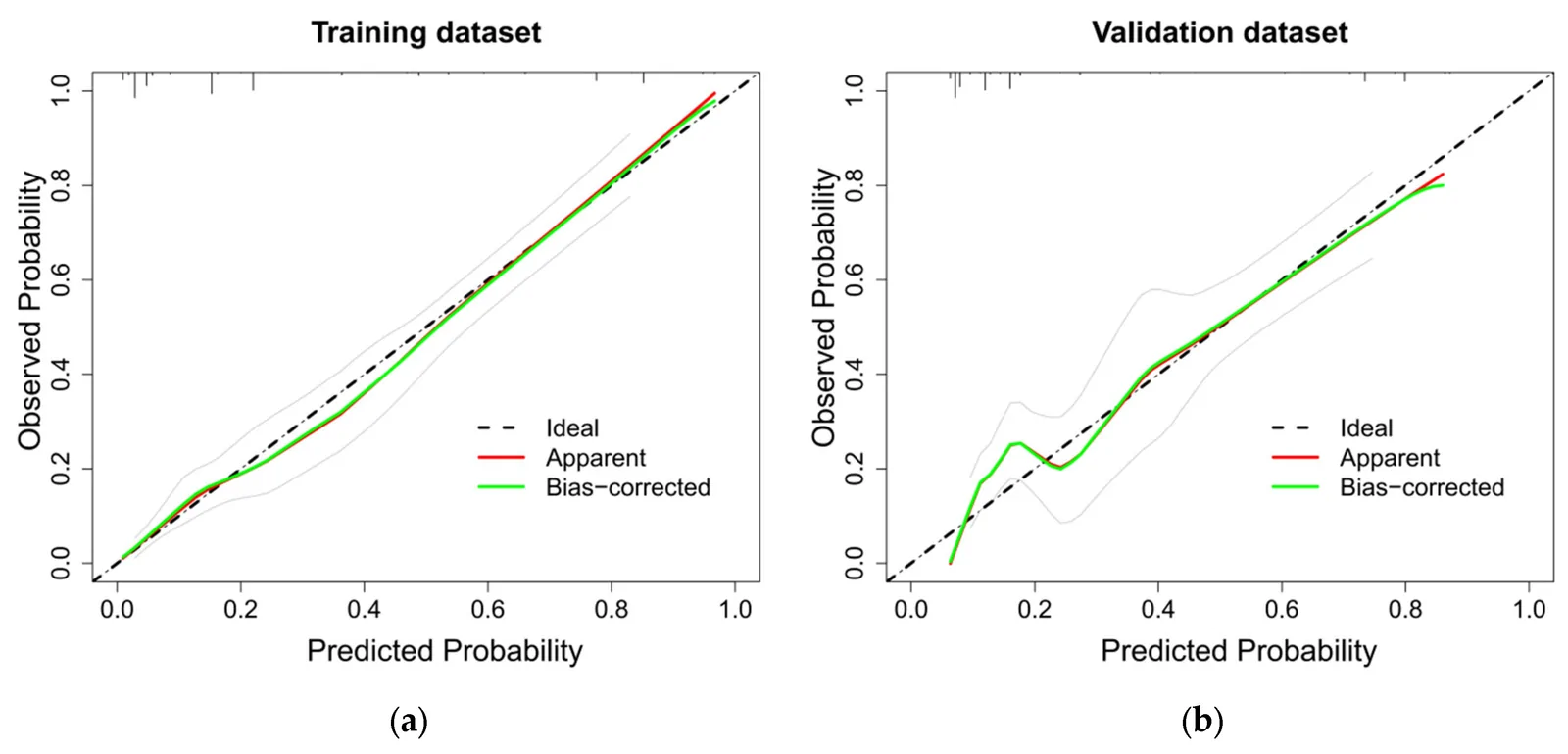
**The calibration curves of the nomogram in the training and validation datasets.** (**a**) The calibration curve in the training dataset. (**b**) The calibration curve in the validation dataset.

The calibration curves showed good agreement between predicted and observed probabilities of PsA in both the training (Figure 3a) and temporal–geographic cohorts (Figure 3b). The Hosmer–Lemeshow goodness-of-fit test further confirmed adequate calibration (training cohort: *p* = 0.436; validation cohort: *p* = 0.063), with no significant deviation between predicted and observed probabilities.

#### 3.3.3. Clinical Utility

DCA and CIC were used to assess the nomogram’s clinical utility. In DCA, the *x*-axis represents threshold probability and the *y*-axis denotes net benefit; greater separation between the nomogram (orange curve) and the “treat all” (green) or “treat none” (blue) strategies indicates superior clinical value. In CIC, the *x*-axis shows threshold probability and the *y*-axis displays the number of high-risk individuals; closer alignment between predicted (red line) and observed (blue dashed line) high-risk cases reflects higher predictive accuracy.

**Figure 4:**
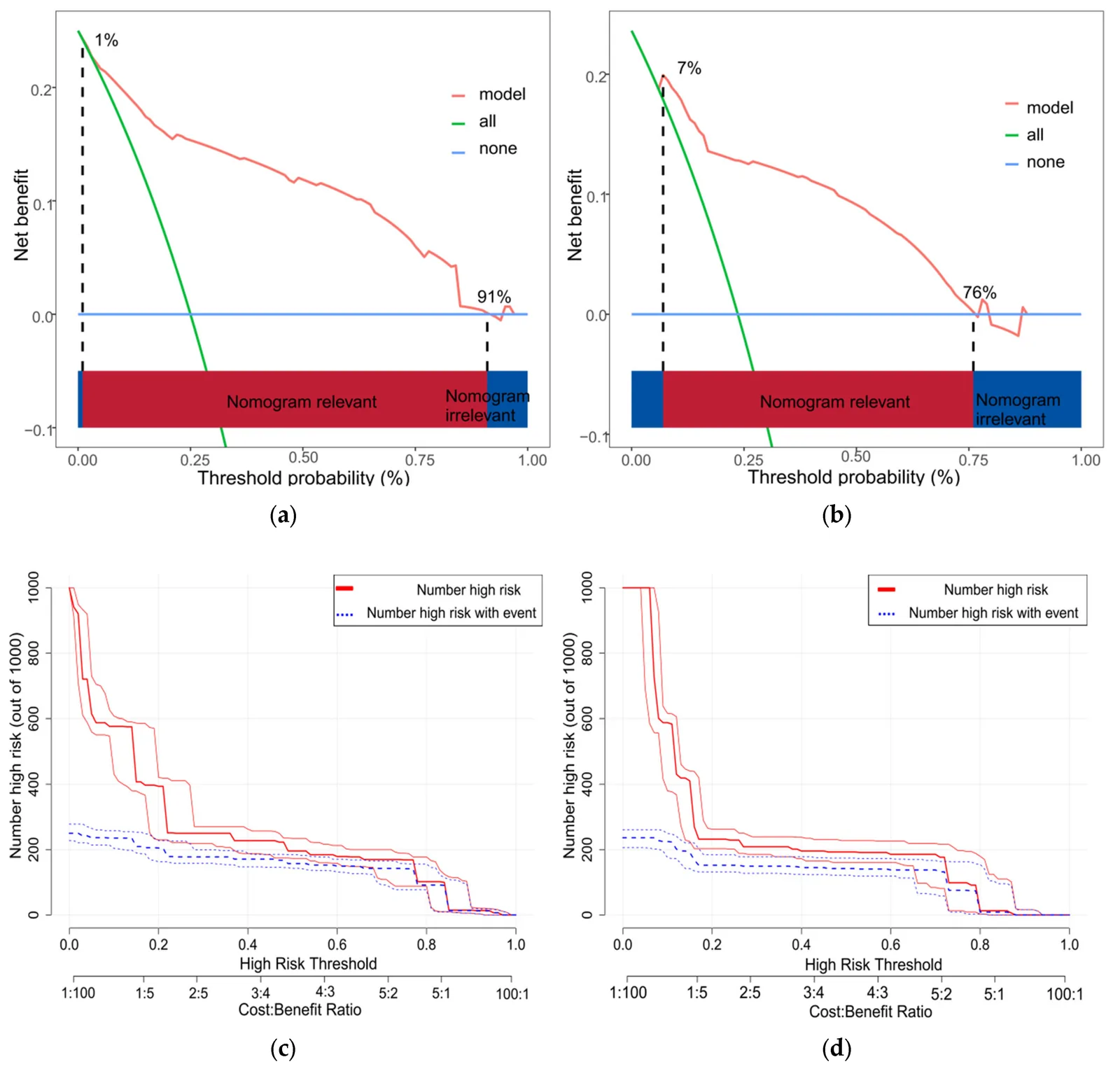
**Clinical utility of the nomogram in the training and validation datasets.** (**a**) The decision curve analysis in the training dataset. (**b**) The decision curve analysis in the validation dataset. (**c**) The clinical impact curve in the training dataset. (**d**) The clinical impact curve in the validation dataset.

DCA showed that the nomogram provided higher net benefit than either extreme strategy across threshold probabilities of 1% to 91% in the training cohort (Figure 4a) and 7% to 76% in temporal–geographic cohort (Figure 4b). At thresholds of 10%, 20%, and 30%, which represent established clinical decision nodes [32], the nomogram avoided 3.1, 9.5, and 21.8 unnecessary referrals per 100 patients, respectively, compared with a ‘refer all’ strategy. Similarly, the CIC showed good alignment between predicted and actual numbers of high-risk patients across clinically relevant threshold probabilities in the training cohort (Figure 4c) and temporal–geographic cohort (Figure 4d), further supporting the model’s clinical applicability.

#### 3.3.4. Sensitivity Analysis

E-value analysis (Table S1, see Supporting Information) demonstrated that the four strongest predictors had E-values (all ≥4.24) substantially exceeding the range of confounding effects typically considered plausible in biomedical research [31]. Inverse lesions showed a more modest E-value.

In addition, a supplementary analysis excluding arthralgia showed that the reduced model, comprising four objective clinical signs, achieved an AUC of 0.759 (95% CI: 0.725–0.793), with a Youden index optimal threshold of 0.247, corresponding to a sensitivity of 84.2% and a specificity of 59.3%. Calibration was excellent (Hosmer–Lemeshow *p* = 0.999), and decision curve analysis confirmed positive net benefit across threshold probabilities of approximately 5% to 81% (Table S2, Figure S4, see Supporting Information).

## 4. Discussion

In this multicenter study, we developed and validated a clinically practical nomogram for estimating PsA probability in psoriasis patients, using a relatively large dataset from two independent centers. The model integrates five key predictors: arthralgia, nail lesions, scalp lesions, inverse lesions and uveitis. Early recognition of PsA may facilitate timely specialist referral and earlier initiation of effective therapy. Recent real-world studies have demonstrated sustained effectiveness of biologics in PsA [33]. Our nomogram was developed and validated in tertiary hospital settings, where the prevalence of PsA may differ from that in general dermatology clinics. Nevertheless, all five predictors are obtainable through routine clinical interview and physical examination, without requiring laboratory tests, imaging, or specialized equipment. This makes the tool potentially adaptable to tertiary dermatology settings where rheumatology specialist access is limited. However, the generalizability of our findings to populations outside tertiary care, particularly those with lower disease severity or different comorbidity profiles, remains to be directly evaluated.

In our model, arthralgia was identified as a key predictor, aligning with the EULAR task force’s characterization of arthralgia as a core feature of subclinical PsA [34,35]. This finding underscores the clinical importance of taking nonspecific joint symptoms seriously in psoriasis patients. However, we acknowledge that arthralgia may carry incorporation bias, as it overlaps with the clinical features that prompted rheumatologists to suspect PsA. To address this concern, we developed a supplementary model excluding arthralgia. This model, comprising only four objective clinical signs (nail, scalp, and inverse lesions, plus uveitis), achieved an AUC of 0.759 (95% CI: 0.725–0.793), with excellent calibration and positive net benefit on DCA. While more modest than the complete model, this performance remains clinically meaningful and suggests that objective signs alone still provide useful discriminative information. We therefore propose a complementary role for the two models: the complete model prioritizes sensitivity for symptomatic patients in dermatology clinics, while the model excluding arthralgia offers a more objective alternative for asymptomatic screening or when joint symptoms are ambiguous. The strength of our nomogram lies in this flexible two-step approach, combining arthralgia as a sensitive ‘red flag’ with objective dermatological signs to improve specificity, helping clinicians identify which patients with nonspecific joint symptoms most likely benefit from specialist referral.

The association between nail lesions and PsA can be explained by the anatomical link between the nail matrix and the distal interphalangeal enthesis, where inflammation at one site may propagate to the other [36]. Scalp lesions are consistent with a population-based cohort that reported a hazard ratio (HR) of 3.75 for PsA development [8]; we consider scalp involvement a useful clinical red flag, though its mechanistic independence from other cutaneous features remains unclear. Uveitis is a recognized extra-articular manifestation of PsA [37]. However, given its low prevalence in our non-PsA group (1.5%) and its dependence on ophthalmologist confirmation, we suggest it be used as a supplemental history item rather than a core screening criterion. Regarding inverse lesions, unlike nail or scalp lesions, the biological link between intertriginous psoriasis and arthritis is less well defined [38]. We speculate that this association may reflect a more aggressive psoriatic phenotype, higher systemic inflammatory burden, or shared environmental triggers—an important question for future research.

Unlike existing screening tools such as PASE [13] and ToPAS [14], which rely on patient recall of symptoms like “swollen joints,” our model uses physician-assessed arthralgia, visual inspection of nail, scalp, and inverse lesions, and verified uveitis history, all of which are readily obtainable. This eliminates the need for laboratory tests, imaging, or specialized equipment, making the tool feasible for rapid implementation in dermatology settings. Building on this practical design, the nomogram further translates a complex regression model into an intuitive point-based score, allowing clinicians to calculate an individualized probability of PsA and make data-driven referral decisions.

Compared with existing models, our nomogram demonstrated competitive discriminative performance. The PRESTO model [39] reported modest discrimination (AUC 0.723–0.749). Zhan et al. [17] achieved an AUC of 0.781. Notably, our model excluding arthralgia achieved an AUC of 0.759, which is comparable to or better than some existing instruments, while the complete model further improves sensitivity by incorporating arthralgia as a red-flag symptom. This flexibility, offering either a signs-only or a complete version depending on the clinical context, provides a pragmatic and scalable solution that directly addresses the major barrier of delayed PsA diagnosis and fulfils the 2023 GRAPPA call for simple, pragmatic early identification instruments [40].

Several limitations should be acknowledged. The cross-sectional design prevents establishing temporal relationships between clinical features and PsA diagnosis. Several potential confounders were not available in our dataset, including Psoriasis Area and Severity Index (PASI), treatment history, psoriasis subtype, dactylitis, and enthesitis. While we acknowledge that the omission of these variables may have introduced residual confounding, our E-value analysis suggested that such confounding is unlikely to fully explain the core findings. The validation cohort was independent from the derivation cohort in terms of recruitment site and period. However, both centers are tertiary hospitals located in the same city, with overlapping recruitment windows. Therefore, this represents a temporal and geographic validation rather than evidence of generalizability across diverse settings. The applicability of our findings to other geographic regions or healthcare systems remains to be established. The exclusion of patients with other musculoskeletal conditions, including osteoarthritis and gout, was necessary for model development but may limit applicability in real-world settings where such comorbidities are common. Verification bias may be present, as all patients in our study underwent rheumatologist assessment.

## 5. Future Directions

Future research should focus on prospective validation of the nomogram in longitudinal cohorts to assess its performance in identifying individuals who will later develop incident PsA. Second, implementation studies are needed to evaluate the nomogram’s impact on referral patterns and clinical outcomes. Third, the development of a web-based or mobile application based on the nomogram could facilitate its widespread adoption in clinical practice.

## 6. Conclusions

In conclusion, the nomogram developed in this study represents a step forward in the clinical identification of psoriasis patients with likely PsA. By integrating easily accessible clinical variables, our model offers a practical and reliable tool for supporting case-finding in tertiary dermatology outpatient care, facilitating timely referral and intervention. Future research should validate the nomogram in diverse populations to further assess its applicability and support broader clinical use.

## Figures and Tables

**Table 1:** Characteristics of patients in the training and validation cohorts.

Variables	Level	Training/Internal Validation Cohort			Temporal–Geographic Cohort		
		Non-PsA (*n* = 663)	PsA (*n* = 221)	*p*-Value	Non-PsA (*n* = 527)	PsA (*n* = 163)	*p*-Value
Sex (%)				0.427			0.042
	Female	204 (30.8)	75 (33.9)		133 (25.2)	55 (33.7)	
	Male	459 (69.2)	146 (66.1)		394 (74.8)	108 (66.3)	
Smoking history (%)				0.433			0.011
	No	438 (66.1)	153 (69.2)		353 (67.0)	127 (77.9)	
	Yes	225 (33.9)	68 (30.8)		174 (33.0)	36 (22.1)	
Educational level (%)				0.472			0.953
	Primary education and below	28 (4.2)	12 (5.4)		24 (4.5)	8 (4.9)	
	Secondary education	149 (22.5)	56 (25.4)		148 (28.1)	44 (27.0)	
	Post-secondary education and above	486 (73.3)	153 (69.2)		355 (67.4)	111 (68.1)	
Family psoriasis history (%)				0.159			0.210
	No	496 (74.8)	154 (69.7)		379 (71.9)	126 (77.3)	
	Yes	167 (25.2)	67 (30.3)		148 (28.1)	37 (22.7)	
Uveitis (%)				<0.001			0.023
	No	653 (98.5)	200 (90.5)		498 (94.5)	145 (89.0)	
	Yes	10 (1.5)	21 (9.5)		29 (5.5)	18 (11.0)	
Inverse lesions (%)				<0.001			<0.001
	No	416 (62.7)	96 (43.4)		330 (62.6)	74 (45.4)	
	Yes	247 (37.3)	125 (56.6)		197 (37.4)	89 (54.6)	
Scalp lesion (%)				<0.001			0.086
	No	117 (17.6)	16 (7.2)		112 (21.3)	24 (14.7)	
	Yes	546 (82.4)	205 (92.8)		415 (78.7)	139 (85.3)	
Nail lesion (%)				<0.001			<0.001
	No	369 (55.7)	34 (15.4)		286 (54.3)	30 (18.4)	
	Yes	294 (44.3)	187 (84.6)		241 (45.7)	133 (81.6)	
Arthralgia (%)				<0.001			<0.001
	No	596 (89.9)	66 (29.9)		465 (88.2)	46 (28.2)	
	Yes	67 (10.1)	155 (70.1)		62 (11.8)	117 (71.8)	
BMI, median(range), kg/m^2^		24 (14.8,45.55)	23.9 (16.9,37.4)	0.623	24.5 (15.9,60.0)	24.5 (15.2,60.0)	0.289
Age, median (range), years		38 (19,87)	44 (21,75)	<0.001	41 (19,82)	44 (20,83)	0.058
Psoriasis duration, median (range), years		12 (0,54)	12 (1,52)	0.144	10 (0,51)	11 (0,40)	0.362
BSA, median (range), %		7 (1,95)	10 (1,90)	0.016	8 (0,98)	8 (0.1,95)	0.680

BMI, body mass index; BSA, body surface area; PsA, psoriatic arthritis; Non-PsA: Patients with psoriasis not diagnosed with psoriatic arthritis.

**Table 2:** Multivariable logistic regression analysis of factors independently associated with PsA in psoriasis patients.

Variables	β	SE	OR	95%CI	Z	*p*
Intercept	−4.662	0.432	0.01	0–0.02	−10.782	<0.001
Uveitis	1.655	0.506	5.23	1.94–14.10	3.271	0.0011
Inverse lesions	0.498	0.21	1.65	1.09–2.48	2.366	0.018
Scalp lesion	1.089	0.367	2.97	1.45–6.10	2.969	0.003
Nail lesion	1.788	0.246	5.98	3.69–9.68	7.262	<0.001
Arthralgia	3.007	0.22	20.23	13.14–31.13	13.678	<0.001

β, regression coefficient; SE, standard error; OR, odds ratio; CI, confidence interval.

## Data Availability

The de-identified training and validation datasets supporting the findings of this study are available in the Supporting Information (Training dataset and Validation dataset). These files include all variables used in model development and temporal–geographic, with patient identifiers removed to ensure confidentiality.

## Supplementary Materials

The following supporting information can be downloaded at: https://www.mdpi.com/article/10.3390/jcm15166215/s1, File S1. TRIPOD checklist. Completed TRIPOD (Transparent Reporting of a multivariable prediction model for Individual Prognosis or Diagnosis) checklist for this study. File S2. Sample size calculation details. File S3. R package details. This file lists all R packages used in the analysis. Table S1: E-value analysis for unmeasured confounding; Table S2: Multivariable logistic regression analysis of the model excluding arthralgia. Figure S1: Flow diagram of study population; Figure S2: LASSO regression for predictor selection; Figure S3: Internal validation of the nomogram using 1000 bootstrap resamples; Figure S4: Performance of the model excluding arthralgia.

## Abbreviations

PsA	Psoriatic arthritis
Non-PsA	Patients with psoriasis not diagnosed with psoriatic arthritis
AUC	Area under the curve
DCA	Decision curve analysis
CIC	Clinical impact curve
CASPAR	Classification criteria for psoriatic arthritis
TRIPOD	Transparent Reporting of a Multivariable Prediction Model for Individual Prognosis or Diagnosis
EDC	Electronic data capture
BMI	Body mass index
BSA	Body surface area
SD	Standard deviation
ROC	Receiver operating characteristic
Β	Regression coefficient
SE	Standard error
OR	Odds ratio
HR	Hazard ratio
GWAS	Genome-Wide Association Study
GRAPPA	Group for Research and Assessment of Psoriasis and Psoriatic Arthritis
PASI	Psoriasis Area and Severity Index
