# Supplementary material for: A Clinically Accessible Nomogram for Identifying Likely Psoriatic Arthritis Among Patients with Psoriasis: A Multicenter Cross-Sectional Study
Source: J Clin Med. 2026 Aug 11;15(16):6215. doi: 10.3390/jcm15166215 (PMC13512928; doi:10.3390/jcm15166215)
Supplement: Supplementary file 1 [file jcm-15-06215-s001.zip › File S2.pdf]

File S2: Sample size calculation details

Training set sample size

According to Riley and colleagues provide guidance and Code on sample size calculations for developing a clinical prediction model, We assumed that cstatistic is 0.75, shrinkage is 0.9,so the pmsampsize code is:pmsampsize(type = "b", parameters = 13, prevalence = 0.2, cstatistic = 0.75, shrinkage = 0.9, seed = 123456),this calculation suggests that minimum sample size required is 866.

Internal validation set sample size

Collins and colleagues suggested using all available data to construct the model, bootstrapping is recommended for internal validation,so the internal validation sample size is equal Training set sample size.

Temporal-geographic validation set sample size

Also according to Snell [25] and Riley [26], guidance and code for sample size calculations for an temporal-geographic validation study are provided. Taking into account both the precision requirements of the study and practical feasibility, we assumed that lpbeta1 is 1.33, lpbeta2 is 1.75, oeciwidth is 0.3, csciwidth is 0.4, and cstatciwidth is 0.15.so the pmvalsampsize code is:pmvalsampsize(type = "b",prevalence =0.2 , cstatistic= 0.75, lpbeta1= 1.33, lpbeta2= 1.75, oeciwidth =0.3, csciwidth=0.4, cstatciwidth=0.15),this calculation suggests that minimum sample size required is 688.
