# Supplementary material for: A Clinically Accessible Nomogram for Identifying Likely Psoriatic Arthritis Among Patients with Psoriasis: A Multicenter Cross-Sectional Study
Source: J Clin Med. 2026 Aug 11;15(16):6215. doi: 10.3390/jcm15166215 (PMC13512928; doi:10.3390/jcm15166215)
Supplement: Supplementary file 1 [file jcm-15-06215-s001.zip › File S3.pdf]

File S3:Detailed information on the R packages

Model Development Code

```
setwd("C:/Users/GUO/Desktop")
library(readr)
library(mice)

dev <- read_csv("Training dataset.csv", show_col_types = FALSE)
dev <- as.data.frame(dev)

cat("=== Missing data summary ===\n")
missing_pct <- colMeans(is.na(dev)) * 100
print(round(missing_pct, 2))

if (any(missing_pct > 0 & missing_pct < 10)) {
  dev_imp <- mice(dev, m = 5, seed = 123, printFlag = FALSE)
  dev <- complete(dev_imp)
  cat("\n=== Multiple imputation completed ===\n")
} else {
  cat("\n=== No missing data, skip imputation ===\n")
}
for(i in names(dev)[c(1:10)]) {
  dev[, i] <- as.factor(dev[, i])
}

dev$PsA <- as.factor(dev$PsA)

# ============================================================
# Descriptive statistics with appropriate tests
# ============================================================

continuous_vars <- c("Age", "Psoriasis_duration", "BSA", "BMI")
categorical_vars <- c("Sex", "Smoking_history", "Educational_Level",
                      "Family_psoriasis_history", "Uveitis", "Inverse_lesions",
                      "Scalp_lesions", "Nail_lesions", "Arthralgia")
```

```r
PsA_0_desc <- c()
PsA_1_desc <- c()
p_values <- c()
test_methods <- c()

for (var in continuous_vars) {
  group0 <- dev[dev$PsA == 0, var]
  group1 <- dev[dev$PsA == 1, var]

  ks0 <- ks.test(group0, "pnorm", mean = mean(group0), sd = sd(group0))
  ks1 <- ks.test(group1, "pnorm", mean = mean(group1), sd = sd(group1))



  if (ks0$p.value > 0.05 & ks1$p.value > 0.05) {
    mean0 <- mean(group0)
    sd0 <- sd(group0)
    mean1 <- mean(group1)
    sd1 <- sd(group1)

    PsA_0_desc[var] <- paste0(round(mean0, 2), " ± ", round(sd0, 2))
    PsA_1_desc[var] <- paste0(round(mean1, 2), " ± ", round(sd1, 2))

    tt <- t.test(group0, group1)
    p_values[var] <- tt$p.value
    test_methods[var] <- "Student's t-test"
  } else {

    med0 <- median(group0)
    min0 <- min(group0)
    max0 <- max(group0)
    med1 <- median(group1)
    min1 <- min(group1)
    max1 <- max(group1)
```

```
    PsA_0_desc[var] <- paste0(med0, " (", min0, ",", max0, ")")
    PsA_1_desc[var] <- paste0(med1, " (", min1, ",", max1, ")")

    wt <- wilcox.test(group0, group1)
    p_values[var] <- wt$p.value
    test_methods[var] <- "Mann-Whitney U test"
  }
}
for (var in categorical_vars) {
  tab <- table(dev[, var], dev$PsA)
  total0 <- sum(tab[, "0"])
  total1 <- sum(tab[, "1"])

  desc0 <- c()
  desc1 <- c()
  for (i in 1:nrow(tab)) {
    level <- rownames(tab)[i]
    n0 <- tab[i, "0"]
    n1 <- tab[i, "1"]
    pct0 <- round(n0 / total0 * 100, 1)
    pct1 <- round(n1 / total1 * 100, 1)
    desc0 <- c(desc0, paste0(level, ": ", n0, " (", pct0, "%)"))
    desc1 <- c(desc1, paste0(level, ": ", n1, " (", pct1, "%)"))
  }
  PsA_0_desc[var] <- paste(desc0, collapse = "; ")
  PsA_1_desc[var] <- paste(desc1, collapse = "; ")

  expected <- chisq.test(tab)$expected
  if (min(expected) < 5) {
    ft <- fisher.test(tab)
    p_values[var] <- ft$p.value
    test_methods[var] <- "Fisher's exact"
  } else {
    ct <- chisq.test(tab)
```

```
    p_values[var] <- ct$p.value
    test_methods[var] <- "Chi-square"
  }
}

results_df <- data.frame(
  Variable = c(continuous_vars, categorical_vars),
  PsA_0 = PsA_0_desc,
  PsA_1 = PsA_1_desc,
  Test_method = test_methods,
  P_value = round(p_values, 4)
)

write.csv(results_df, "Descriptive_Statistics_with_Percentages.csv", row.names =
FALSE)
print(results_df)

# ========== LASSO ==========
library(glmnet)
library(ggplot2)
library(reshape2)

vars <- c("Sex", "Smoking_history", "Educational_Level", "Family_psoriasis_history",
          "Uveitis", "Inverse_lesions", "Scalp_lesions", "Nail_lesions",
          "Arthralgia", "BMI", "Age", "Psoriasis_duration", "BSA")
x_factor <- model.matrix(~ . - 1, data = dev[, vars])
y <- dev$PsA

set.seed(123)
cv_lasso <- cv.glmnet(x_factor, y, family = "binomial", alpha = 1)

png("LASSO_CrossValidation.png", width = 3000, height = 2000, res = 300)
plot(log(cv_lasso$lambda), cv_lasso$cvm, pch = 20, col = "red", cex = 1.2,
     xlab = expression(log(lambda)), ylab = "Binomial Deviance", main = "")
```

```
arrows(log(cv_lasso$lambda), cv_lasso$cvlo, log(cv_lasso$lambda), cv_lasso$cvup,
       length = 0.05, angle = 90, code = 3, col = "gray80", lwd = 1.5)
abline(v = log(cv_lasso$lambda.min), col = "blue", lty = 2, lwd = 2)
abline(v = log(cv_lasso$lambda.1se), col = "green", lty = 2, lwd = 2)
axis(3, at = log(cv_lasso$lambda)[seq(1, length(cv_lasso$lambda), length.out = 10)],
     labels = cv_lasso$nzero[seq(1, length(cv_lasso$lambda), length.out = 10)], cex.axis
= 0.9)
legend("topright", legend = c("lambda.min", "lambda.1se"),
       col = c("blue", "green"), lty = 2, lwd = 2, cex = 1.2, bty = "n")
dev.off()

full_lasso <- glmnet(x_factor, y, family = "binomial", alpha = 1)
path_data <- as.data.frame(as.matrix(full_lasso$beta))
path_data$var <- rownames(path_data)
path_long <- melt(path_data, id.vars = "var", variable.name = "idx", value.name =
"coef")
path_long$log_lambda <- log(full_lasso$lambda)[as.numeric(path_long$idx)]
keep <- names(which(tapply(abs(path_long$coef), path_long$var, max) > 0.05))
plot_data <- path_long[path_long$var %in% keep, ]

png("LASSO_Coefficient_Path.png", width = 3500, height = 2500, res = 300)
ggplot(plot_data, aes(x = log_lambda, y = coef, color = var, group = var)) +
  geom_line(linewidth = 1.2, alpha = 0.8) +
  geom_vline(xintercept = log(cv_lasso$lambda.min), linetype = "dashed", color =
"blue", linewidth = 1) +
  geom_vline(xintercept = log(cv_lasso$lambda.1se), linetype = "dashed", color =
"green", linewidth = 1) +
  labs(x = expression(log(lambda)), y = "Coefficients", color = NULL) +
  theme_minimal(base_size = 14) +
  theme(legend.position = "right", legend.text = element_text(size = 10),
        axis.title = element_text(size = 14), axis.text = element_text(size = 12),
        panel.grid.minor = element_blank(), panel.grid.major = element_line(color =
"gray90", linewidth = 0.5))
dev.off()
```

```
lambda_min <- cv_lasso$lambda.min
lambda_1se <- cv_lasso$lambda.1se

cat("\n========== lambda.min ==========\n")
cat("lambda value:", lambda_min, "\n")
lasso_min <- glmnet(x_factor, y, family = "binomial", alpha = 1, lambda = lambda_min)
coef_min <- as.matrix(coef(lasso_min))
selected_min <- rownames(coef_min)[coef_min[, 1] != 0]
selected_min <- selected_min[selected_min != "(Intercept)"]
cat("Number of variables:", length(selected_min), "\n")
print(selected_min)

cat("\n========== lambda.1se ==========\n")
cat("lambda value:", lambda_1se, "\n")
lasso_1se <- glmnet(x_factor, y, family = "binomial", alpha = 1, lambda = lambda_1se)
coef_1se <- as.matrix(coef(lasso_1se))
selected_1se <- rownames(coef_1se)[coef_1se[, 1] != 0]
selected_1se <- selected_1se[selected_1se != "(Intercept)"]
cat("Number of variables:", length(selected_1se), "\n")
print(selected_1se)

# Multivariate logistic regression
five_vars <- c("Arthralgia", "Nail_lesions", "Uveitis", "Scalp_lesions", "Inverse_lesions")
fml <- as.formula(paste("PsA ~", paste(five_vars, collapse = " + ")))
model <- glm(fml, data = dev, family = binomial)

glm_sum <- summary(model)
OR <- round(exp(coef(model)), 2)
SE <- round(glm_sum$coefficients[, 2], 3)
CI2.5 <- round(exp(coef(model) - 1.96 * SE), 2)
CI97.5 <- round(exp(coef(model) + 1.96 * SE), 2)
CI <- paste0(CI2.5, "-", CI97.5)
B <- round(coef(model), 3)
```

```
Z <- round(glm_sum$coefficients[, 3], 3)
P <- round(glm_sum$coefficients[, 4], 4)

mlogit <- data.frame('B' = B, 'SE' = SE, 'OR' = OR, 'CI' = CI, 'Z' = Z, 'P' = P)
mlogit <- data.frame('characteristics' = rownames(mlogit), mlogit)
write.csv(mlogit, "Multivariate_Analysis_Results.csv", row.names = FALSE)

# VIF
library(car)
model_vif <- glm(PsA ~ Arthralgia + Nail_lesions + Scalp_lesions + Inverse_lesions +
Uveitis,
                 data = dev, family = binomial)
vif_results <- vif(model_vif)
print(vif_results)
final_model <- glm(PsA ~ Arthralgia + Nail_lesions + Scalp_lesions +
                   Inverse_lesions + Uveitis,
                 data = dev, family = binomial)

cat("\n========== Final Model Coefficients ==========\n")
print(coef(final_model))

saveRDS(final_model, "training_model.rds")
cat("\nModel saved to: C:/Users/GUO/Desktop/training_model.rds\n")



# Nomogram
library(rms)
dev$Arthralgia <- factor(dev$Arthralgia, levels = c(0, 1), labels = c("No", "Yes"))
dev$Nail_lesions <- factor(dev$Nail_lesions, levels = c(0, 1), labels = c("No", "Yes"))
dev$Scalp_lesions <- factor(dev$Scalp_lesions, levels = c(0, 1), labels = c("No", "Yes"))
dev$Inverse_lesions <- factor(dev$Inverse_lesions, levels = c(0, 1), labels = c("No",
"Yes"))
dev$Uveitis <- factor(dev$Uveitis, levels = c(0, 1), labels = c("No", "Yes"))
ddist <- datadist(dev)
```

```
options(datadist = 'ddist')
model_nomo <- lrm(PsA ~ Arthralgia + Nail_lesions + Scalp_lesions + Inverse_lesions
+ Uveitis, data = dev)
nomomodel <- nomogram(model_nomo, lp = FALSE, fun = function(x) 1/(1+exp(-x)),
                fun.at = seq(0.1, 0.9, by = 0.1), funlabel = "Diagnostic Probability")
png("Nomogram.png", width = 4000, height = 2500, res = 300)
plot(nomomodel, col.grid = c("tomato", "darkcyan"))
dev.off()

# ROC curve
fml_psa <- as.formula(PsA == 1 ~ Arthralgia + Nail_lesions + Scalp_lesions +
Inverse_lesions + Uveitis)
model_roc <- glm(fml_psa, data = dev, family = binomial(logit))
dev$pred_psa <- predict(model_roc, type = "response")
library(pROC)
roc_dev <- roc(dev$PsA, dev$pred_psa, ci = TRUE)
auc_dev <- auc(roc_dev)

cat("AUC =", round(auc_dev, 3), "\n")
cat("95% CI:", round(ci.auc(roc_dev)[1], 3), "-", round(ci.auc(roc_dev)[3], 3), "\n")

youden_idx <- which.max(roc_dev$sensitivities + roc_dev$specificities - 1)
best_threshold <- roc_dev$thresholds[youden_idx]
best_sensitivity <- roc_dev$sensitivities[youden_idx]
best_specificity <- roc_dev$specificities[youden_idx]

cat("\n=== Optimal Threshold (Youden Index) ===\n")
cat("Best threshold:", round(best_threshold, 3), "\n")
cat("Sensitivity:", round(best_sensitivity, 3), "\n")
cat("Specificity:", round(best_specificity, 3), "\n")

png("ROC_Curve.png", width = 3500, height = 2800, res = 300)
plot(roc_dev, print.auc = TRUE, auc.polygon = TRUE, max.auc.polygon = TRUE,
   auc.polygon.col = "skyblue", print.thres = "best",
```

```
    main = "Training dataset",
    col = "red", print.thres.col = "black",
    identity.col = "blue", identity.lty = 2)
dev.off()

# ================================================================
# Sensitivity analysis: model without arthralgia
# ================================================================

library(pROC)

# Model B: remove arthralgia
vars_b <- c("Nail_lesions", "Scalp_lesions", "Inverse_lesions", "Uveitis")
fml_b <- as.formula(paste("PsA ~", paste(vars_b, collapse = " + ")))

model_b <- glm(fml_b, data = dev, family = binomial)
dev$pred_b <- predict(model_b, type = "response")
roc_b <- roc(dev$PsA, dev$pred_b, quiet = TRUE)
auc_b <- auc(roc_b)

cat("Model without arthralgia AUC:", round(auc_b, 3), "\n")
cat("95% CI:", round(ci.auc(roc_b)[1], 3), "-", round(ci.auc(roc_b)[3], 3), "\n")

# ================================================================
# Subgroup analysis by sex and age groups
# ================================================================

library(pROC)

vars <- c("Arthralgia", "Nail_lesions", "Scalp_lesions", "Inverse_lesions", "Uveitis")
fml <- as.formula(paste("PsA ~", paste(vars, collapse = " + ")))

# Define subgroups
subgroups <- list(
```

```
    "Female" = dev[dev$Sex == 0, ],
    "Male" = dev[dev$Sex == 1, ],
    "Age <40" = dev[dev$Age < 40, ],
    "Age 40-60" = dev[dev$Age >= 40 & dev$Age <= 60, ],
    "Age >60" = dev[dev$Age > 60, ]
  )

  # Store results
  subgroup_results <- data.frame(Subgroup = character(), AUC = numeric(), N =
integer(), PsA_N = integer())

  for (name in names(subgroups)) {
    data <- subgroups[[name]]
    n_total <- nrow(data)
    n_psa <- sum(data$PsA == 1)

    # Check if sample size is sufficient (at least 30 cases, and both PsA and non-PsA
have at least 5 cases)
    if (n_total >= 30 & sum(data$PsA == 1) >= 5 & sum(data$PsA == 0) >= 5) {
      model <- glm(fml, data = data, family = binomial)
      data$pred <- predict(model, type = "response")
      roc_obj <- roc(data$PsA, data$pred, quiet = TRUE)
      auc_val <- round(auc(roc_obj), 3)
    } else {
      auc_val <- NA
    }

    subgroup_results <- rbind(subgroup_results,
                              data.frame(Subgroup = name,
                                         AUC = auc_val,
                                         N = n_total,
                                         PsA_N = n_psa))
  }
```

```
print(subgroup_results)
write.csv(subgroup_results, "Subgroup_AUC_Results.csv", row.names = FALSE)

cat("\n=== Subgroup Analysis Results ===\n")
print(subgroup_results)

# Bootstrap ROC curves (1000 times)
set.seed(123)
labels <- dev$PsA
pred <- dev$pred_psa
n_bootstraps <- 1000
roc_boot <- list()

for (i in 1:n_bootstraps) {
  boot_indices <- sample(length(pred), replace = TRUE)
  boot_pred <- pred[boot_indices]
  boot_labels <- labels[boot_indices]
  roc_boot[[i]] <- roc(boot_labels, boot_pred, quiet = TRUE)
}

png("ROC_Curve_Bootstrap.png", width = 3500, height = 2800, res = 300)
plot(roc_boot[[1]], type = "n",
     main = "Internal Validation (1000 Bootstraps)",
     xlab = "False Positive Rate", ylab = "True Positive Rate")
for (i in 1:n_bootstraps) {
  lines(roc_boot[[i]], col = "grey", alpha = 0.2)
}
roc_mean <- roc(labels, pred, ci = TRUE)
lines(roc_mean, col = "red", lwd = 3)
legend("bottomright", legend = c("Bootstrap ROC", "Mean ROC"),
       col = c("grey", "red"), lwd = c(1, 3), bty = "n")
dev.off()

# Bootstrap statistics
```

```
auc_values <- sapply(roc_boot, function(x) auc(x))
auc_mean <- mean(auc_values)
auc_sd <- sd(auc_values)
auc_ci <- quantile(auc_values, c(0.025, 0.975))

cat("\n=== Bootstrap Validation (1000次) ===\n")
cat("Bootstrap Mean AUC:", round(auc_mean, 3), "\n")
cat("Bootstrap 95% CI:", round(auc_ci[1], 3), "-", round(auc_ci[2], 3), "\n")
cat("Bootstrap SD:", round(auc_sd, 4), "\n")

# Calibration curve (rms)
library(rms)
model_cal <- lrm(PsA ~ Arthralgia + Nail_lesions + Scalp_lesions + Inverse_lesions +
Uveitis,
                 data = dev, x = TRUE, y = TRUE)
cal_train <- calibrate(model_cal, method = 'boot', m = 50, B = 1000)
png("Calibration_Curve.png", width = 3500, height = 2800, res = 300)
plot(cal_train, xlim = c(0, 1), ylim = c(0, 1), xlab = "Predicted Probability",
   ylab = "Observed Probability", legend = FALSE, main = "Training dataset", subtitles
= FALSE)
abline(0, 1, col = "black", lty = 2, lwd = 2)
lines(cal_train[, c("predy", "calibrated.orig")], type = "l", lwd = 2, col = "red")
lines(cal_train[, c("predy", "calibrated.corrected")], type = "l", lwd = 2, col = "green")
legend(0.55, 0.35, c("Ideal", "Apparent", "Bias-corrected"), lty = c(2, 1, 1),
    lwd = c(2, 2, 2), col = c("black", "red", "green"), bty = "n")
dev.off()

# Calibration with riskRegression
library(riskRegression)
formula_psa <- PsA == 1 ~ Arthralgia + Nail_lesions + Scalp_lesions + Inverse_lesions
+ Uveitis
fit_psa <- glm(formula_psa, data = dev, family = binomial())
xb_psa <- Score(list("5-variable model" = fit_psa), formula = PsA ~ 1,
```

```
              null.model = FALSE, plots = c("calibration"), metrics = c("brier"),
              B = 1000, M = 50, data = dev)
png("Calibration_Curve_Score.png", width = 3500, height = 2800, res = 300)
plotCalibration(xb_psa, col = "red", xlab = "Predicted Probability",
              ylab = "Observed Probability", main = "PsA Model Calibration")
dev.off()

# Hosmer-Lemeshow test
library(ResourceSelection)
dev$PsA_num <- as.numeric(dev$PsA) - 1
dev$pred_psa_prob <- predict(fit_psa, type = "response")
if(nrow(dev) >= 100) {
  hl_test <- hoslem.test(dev$PsA_num, dev$pred_psa_prob, g = 10)
  cat("Hosmer-Lemeshow test P-value:", round(hl_test$p.value, 4), "\n")

  hl_df <- data.frame(
    Test = "Hosmer-Lemeshow",
    Groups = 10,
    Chi_square = round(hl_test$statistic, 3),
    DF = hl_test$parameter,
    P_value = round(hl_test$p.value, 4)
  )
  write.csv(hl_df, "HLtest_Result.csv", row.names = FALSE)
} else {
  cat("Sample size (n=", nrow(dev), ") is too small for HL test (need >=100)\n")
}


# DCA
library(ggscidca)

dev$PsA_num <- as.numeric(dev$PsA) - 1

dca_glm <- glm(PsA_num ~ Arthralgia + Nail_lesions + Scalp_lesions +
```

```
Inverse_lesions + Uveitis,
              data = dev, family = binomial(link = "logit"))

    png("DCA.png", width = 3500, height = 2800, res = 300)
    scidca(dca_glm,
        threshold.line = TRUE,
        threshold.text = TRUE,
        text.size = 4,
        text.col = "black")
    dev.off()

    # ============================================================
    # Calculate clinical impact: unnecessary referrals avoided
    # ============================================================

    library(rmda)

    # Fit decision curve (if not already fitted)
    dca_fit <- decision_curve(
     PsA_num ~ Arthralgia + Nail_lesions + Scalp_lesions + Inverse_lesions + Uveitis,
     data = dev,
     family = binomial(link = 'logit'),
     thresholds = seq(0, 1, by = 0.01),
     bootstraps = 100
    )

    # Extract net benefit at clinically relevant thresholds
    thresholds_of_interest <- c(0.1, 0.2, 0.3)
    nb_model <- c()
    nb_all <- c()
    nb_none <- c()

    for(i in 1:length(thresholds_of_interest)) {
     idx <- which.min(abs(dca_fit$derived.data$thresholds - thresholds_of_interest[i]))
```

```
  nb_model[i] <- dca_fit$derived.data$NB[idx]
  nb_all[i] <- dca_fit$derived.data$NB[idx + nrow(dca_fit$derived.data)/3]  # All strategy
  nb_none[i] <- 0  # None strategy always 0
}

# Calculate unnecessary referrals avoided per 100 patients
avoided_per_100 <- round((nb_model - nb_all) * 100, 1)

# Create results table
impact_table <- data.frame(
  Threshold = c("10%", "20%", "30%"),
  Net_Benefit_Model = round(nb_model, 4),
  Net_Benefit_All = round(nb_all, 4),
  Avoided_Referrals_per_100 = avoided_per_100
)

print(impact_table)
write.csv(impact_table, "Clinical_Impact_Estimates.csv", row.names = FALSE)

cat("\n=== Clinical Impact Estimates ===\n")
cat("At 10% threshold: avoid", avoided_per_100[1], "unnecessary referrals per 100
patients\n")
cat("At 20% threshold: avoid", avoided_per_100[2], "unnecessary referrals per 100
patients\n")
cat("At 30% threshold: avoid", avoided_per_100[3], "unnecessary referrals per 100
patients\n")

# CIC
library(rmda)
dev$PsA_num <- as.numeric(dev$PsA) - 1
dca_fit <- decision_curve(PsA_num ~ Arthralgia + Nail_lesions + Scalp_lesions +
Inverse_lesions + Uveitis,
                  data = dev, family = binomial(link = 'logit'),
                  thresholds = seq(0, 1, by = 0.01), bootstraps = 50, confidence.intervals
```

```
= 0.95)
      png("Clinical_Impact_Curve.png", width = 4000, height = 3000, res = 300)
      plot_clinical_impact(dca_fit, population.size = 1000, cost.benefit.axis = TRUE,
                  n.cost.benefits = 8, col = c('red', 'blue'), confidence.intervals = TRUE,
                  ylim = c(0, 1000), legend.position = "topright")
      dev.off()

      # Confusion matrix
      library(caret)
      library(reshape2)
      library(ggplot2)
      dev$PsA_num <- as.numeric(dev$PsA) - 1
      model_cm <- glm(PsA_num ~ Arthralgia + Nail_lesions + Scalp_lesions +
Inverse_lesions + Uveitis,
               data = dev, family = binomial(logit))
      dev$predmodel <- predict(model_cm, type = "response")
      predictions <- ifelse(dev$predmodel > 0.5, 1, 0)
      actual <- factor(dev$PsA_num, levels = c(0, 1))
      predictions <- factor(predictions, levels = c(0, 1))
      conf_matrix <- confusionMatrix(predictions, actual)
      print(conf_matrix)

      conf_stats <- data.frame(
        Metric = c("Accuracy", "Sensitivity", "Specificity", "PPV", "NPV", "F1 Score"),
        Value = c(conf_matrix$overall["Accuracy"], conf_matrix$byClass["Sensitivity"],
             conf_matrix$byClass["Specificity"], conf_matrix$byClass["Pos Pred Value"],
             conf_matrix$byClass["Neg Pred Value"], conf_matrix$byClass["F1"])
      )
      write.csv(conf_stats, "Confusion_Matrix_Statistics.csv", row.names = FALSE)

      conf_df <- as.data.frame(conf_matrix$table)
      colnames(conf_df) <- c("Prediction", "Reference", "Count")
      conf_df$Prediction <- factor(conf_df$Prediction, levels = c(0, 1), labels = c("No PsA",
"PsA"))
```

```r
conf_df$Reference <- factor(conf_df$Reference, levels = c(0, 1), labels = c("No PsA", "PsA"))

png("Confusion_Matrix.png", width = 3500, height = 3000, res = 300)
ggplot(conf_df, aes(x = Reference, y = Prediction, fill = Count)) +
  geom_tile(color = "white", linewidth = 1) +
  geom_text(aes(label = Count), color = "white", size = 6, fontface = "bold") +
  scale_fill_gradient(low = "steelblue", high = "darkred", name = "Count") +
  theme_minimal(base_size = 14) +
  labs(title = "Training dataset", x = "Actual", y = "Predicted") +
  theme(axis.text = element_text(size = 12, color = "black"), axis.title = element_text(size = 14, face = "bold"),
        plot.title = element_text(size = 16, face = "bold", hjust = 0.5), panel.grid = element_blank())
dev.off()

# ==================================================================
# Export all figures as PDF (vector format for publication)
# ==================================================================

# Figure 1: Nomogram
pdf("Figure1_Nomogram.pdf", width = 12, height = 8)
plot(nomomodel,
     col.grid = c("tomato", "darkcyan"),
     cex.axis = 1.6,
     cex.lab = 1.6,
     fontsize = 13)
dev.off()

# Figure 2: ROC Curves (training and validation combined, or separate)
# Note: If you have separate ROC objects for training and validation, adjust accordingly
# Here assuming roc_dev is training ROC
pdf("Figure2_ROC_Curve_Training.pdf", width = 8, height = 7)
plot(roc_dev, print.auc = TRUE, auc.polygon = TRUE, max.auc.polygon = TRUE,
```

```
    auc.polygon.col = "skyblue", print.thres = "best",
    main = "Training dataset",
    col = "red", print.thres.col = "black",
    identity.col = "blue", identity.lty = 2,
    cex.main = 1.8, cex.lab = 1.6, cex.axis = 1.5)
dev.off()

# If you have validation ROC (roc_val), uncomment below:
# pdf("Figure2_ROC_Curve_Validation.pdf", width = 8, height = 7)
# plot(roc_val, print.auc = TRUE, auc.polygon = TRUE, max.auc.polygon = TRUE,
#     auc.polygon.col = "skyblue", print.thres = "best",
#     main = "Validation dataset",
#     col = "red", print.thres.col = "black",
#     identity.col = "blue", identity.lty = 2,
#     cex.main = 1.8, cex.lab = 1.6, cex.axis = 1.5)
# dev.off()

# Figure 3: Calibration Curves (training)
pdf("Figure3_Calibration_Curve_Training.pdf", width = 8, height = 7)
plot(cal_train,
    xlim = c(0, 1), ylim = c(0, 1),
    xlab = "Predicted Probability",
    ylab = "Observed Probability",
    legend = FALSE,
    main = "Training dataset",
    subtitles = FALSE,
    cex.main = 1.8, cex.lab = 1.6, cex.axis = 1.5)
abline(0, 1, col = "black", lty = 2, lwd = 3)
lines(cal_train[, c("predy", "calibrated.orig")], type = "l", lwd = 3, col = "red")
lines(cal_train[, c("predy", "calibrated.corrected")], type = "l", lwd = 3, col = "green")
legend(0.55, 0.35,
       c("Ideal", "Apparent", "Bias-corrected"),
       lty = c(2, 1, 1),
       lwd = c(3, 3, 3),
```

```
      col = c("black", "red", "green"),
      bty = "n",
      cex = 1.5)
dev.off()

# Figure 4a: DCA (Training)
pdf("Figure4a_DCA_Training.pdf", width = 8, height = 7)
scidca(dca_glm,
      threshold.line = TRUE,
      threshold.text = TRUE,
      text.size = 5,
      text.col = "black")
dev.off()

# Figure 4b: Clinical Impact Curve (Training)
pdf("Figure4b_Clinical_Impact_Curve_Training.pdf", width = 10, height = 8)
plot_clinical_impact(dca_fit,
                    population.size = 1000,
                    cost.benefit.axis = TRUE,
                    n.cost.benefits = 8,
                    col = c('red', 'blue'),
                    confidence.intervals = TRUE,
                    ylim = c(0, 1000),
                    legend.position = "topright",
                    cex.axis = 1.5,
                    cex.lab = 1.6,
                    cex.main = 1.8)
dev.off()

# Figure 5: Bootstrap ROC (if needed)
pdf("Figure5_Bootstrap_ROC.pdf", width = 8, height = 7)
plot(roc_boot[[1]], type = "n",
    main = "Internal Validation (1000 Bootstraps)",
    xlab = "False Positive Rate", ylab = "True Positive Rate",
```

```
    cex.main = 1.8, cex.lab = 1.6, cex.axis = 1.5)
for (i in 1:n_bootstraps) {
  lines(roc_boot[[i]], col = "grey", alpha = 0.2)
}
lines(roc_mean, col = "red", lwd = 3)
legend("bottomright", legend = c("Bootstrap ROC", "Mean ROC"),
       col = c("grey", "red"), lwd = c(1, 3), bty = "n", cex = 1.4)
dev.off()

cat("\n=== All figures exported as PDF ===\n")
cat("Figure1_Nomogram.pdf\n")
cat("Figure2_ROC_Curve_Training.pdf\n")
cat("Figure3_Calibration_Curve_Training.pdf\n")
cat("Figure4a_DCA_Training.pdf\n")
cat("Figure4b_Clinical_Impact_Curve_Training.pdf\n")
cat("Figure5_Bootstrap_ROC.pdf\n")


# ============================================================
# Sensitivity analysis: model without arthralgia
# ============================================================

library(pROC)
library(rms)
library(ResourceSelection)
library(ggscidca)
library(ggplot2)

cat("\n========== Model without Arthralgia ==========\n")

vars_without_arthralgia <- c("Nail_lesions", "Scalp_lesions", "Inverse_lesions",
"Uveitis")
fml_wo_arthralgia <- as.formula(paste("PsA ~", paste(vars_without_arthralgia, collapse
= " + ")))
```

```
model_wo_arthralgia <- glm(fml_wo_arthralgia, data = dev, family = binomial)
cat("\n=== Model Coefficients ===\n")
print(summary(model_wo_arthralgia))

glm_sum_wo <- summary(model_wo_arthralgia)
OR_wo <- round(exp(coef(model_wo_arthralgia)), 2)
SE_wo <- round(glm_sum_wo$coefficients[, 2], 3)
CI2.5_wo <- round(exp(coef(model_wo_arthralgia) - 1.96 * SE_wo), 2)
CI97.5_wo <- round(exp(coef(model_wo_arthralgia) + 1.96 * SE_wo), 2)
CI_wo <- paste0(CI2.5_wo, "-", CI97.5_wo)
B_wo <- round(coef(model_wo_arthralgia), 3)
Z_wo <- round(glm_sum_wo$coefficients[, 3], 3)
P_wo <- round(glm_sum_wo$coefficients[, 4], 4)

mlogit_wo <- data.frame(
  'characteristics' = rownames(glm_sum_wo$coefficients),
  'B' = B_wo,
  'SE' = SE_wo,
  'OR' = OR_wo,
  'CI' = CI_wo,
  'Z' = Z_wo,
  'P' = P_wo
)
write.csv(mlogit_wo, "Multivariate_Analysis_Results_Without_Arthralgia.csv",
row.names = FALSE)
cat("\n=== Multivariate results saved ===\n")

dev$pred_wo <- predict(model_wo_arthralgia, type = "response")
roc_wo <- roc(dev$PsA, dev$pred_wo, quiet = TRUE)
auc_wo <- auc(roc_wo)
ci_auc_wo <- ci.auc(roc_wo)

cat("\n=== Discrimination (AUC) ===\n")
```

```r
cat("AUC =", round(auc_wo, 3), "\n")
cat("95% CI:", round(ci_auc_wo[1], 3), "-", round(ci_auc_wo[3], 3), "\n")

youden_idx_wo <- which.max(roc_wo$sensitivities + roc_wo$specificities - 1)
best_threshold_wo <- roc_wo$thresholds[youden_idx_wo]
best_sensitivity_wo <- roc_wo$sensitivities[youden_idx_wo]
best_specificity_wo <- roc_wo$specificities[youden_idx_wo]

cat("\n=== Optimal Threshold (Youden Index) ===\n")
cat("Best threshold:", round(best_threshold_wo, 3), "\n")
cat("Sensitivity:", round(best_sensitivity_wo, 3), "\n")
cat("Specificity:", round(best_specificity_wo, 3), "\n")

dev$PsA_num <- as.numeric(dev$PsA) - 1
dev$pred_wo_prob <- predict(model_wo_arthralgia, type = "response")

if(nrow(dev) >= 100) {
  hl_test_wo <- hoslem.test(dev$PsA_num, dev$pred_wo_prob, g = 10)
  cat("\n=== Calibration (Hosmer-Lemeshow) ===\n")
  cat("Chi-square:", round(hl_test_wo$statistic, 3), "\n")
  cat("DF:", hl_test_wo$parameter, "\n")
  cat("P-value:", round(hl_test_wo$p.value, 4), "\n")

  hl_df_wo <- data.frame(
    Model = "Without Arthralgia",
    Test = "Hosmer-Lemeshow",
    Groups = 10,
    Chi_square = round(hl_test_wo$statistic, 3),
    DF = hl_test_wo$parameter,
    P_value = round(hl_test_wo$p.value, 4)
  )
  write.csv(hl_df_wo, "HLtest_Result_Without_Arthralgia.csv", row.names = FALSE)
} else {
  cat("Sample size too small for HL test\n")
```

```
}

cat("\n=== Generating calibration curve ===\n")

dev_rms <- dev
dev_rms$Nail_lesions_num <- as.numeric(dev_rms$Nail_lesions) - 1
dev_rms$Scalp_lesions_num <- as.numeric(dev_rms$Scalp_lesions) - 1
dev_rms$Inverse_lesions_num <- as.numeric(dev_rms$Inverse_lesions) - 1
dev_rms$Uveitis_num <- as.numeric(dev_rms$Uveitis) - 1

model_cal_wo <- lrm(PsA ~ Nail_lesions_num + Scalp_lesions_num +
Inverse_lesions_num + Uveitis_num,
                    data = dev_rms, x = TRUE, y = TRUE)

cal_train_wo <- calibrate(model_cal_wo, method = 'boot', m = 50, B = 1000)

png("Calibration_Curve_Without_Arthralgia.png", width = 3500, height = 2800, res =
300)
plot(cal_train_wo, xlim = c(0, 1), ylim = c(0, 1), xlab = "Predicted Probability",
    ylab = "Observed Probability", legend = FALSE,
    main = "Without Arthralgia (Training)", subtitles = FALSE)
abline(0, 1, col = "black", lty = 2, lwd = 2)
lines(cal_train_wo[, c("predy", "calibrated.orig")], type = "l", lwd = 2, col = "red")
lines(cal_train_wo[, c("predy", "calibrated.corrected")], type = "l", lwd = 2, col = "green")
legend(0.55, 0.35, c("Ideal", "Apparent", "Bias-corrected"), lty = c(2, 1, 1),
       lwd = c(2, 2, 2), col = c("black", "red", "green"), bty = "n")
dev.off()

png("ROC_Curve_Without_Arthralgia.png", width = 3500, height = 2800, res = 300)
plot(roc_wo, print.auc = TRUE, auc.polygon = TRUE, max.auc.polygon = TRUE,
    auc.polygon.col = "skyblue", print.thres = "best",
    main = "Without Arthralgia (Training)",
    col = "red", print.thres.col = "black",
    identity.col = "blue", identity.lty = 2)
```

```
dev.off()

cat("\n=== Generating Decision Curve Analysis ===\n")

dev$PsA_num <- as.numeric(dev$PsA) - 1

dca_glm_wo <- glm(PsA_num ~ Nail_lesions + Scalp_lesions + Inverse_lesions +
Uveitis,
                  data = dev, family = binomial(link = "logit"))

png("DCA_Without_Arthralgia.png", width = 3500, height = 2800, res = 300)
scidca(dca_glm_wo,
       threshold.line = TRUE,
       threshold.text = TRUE,
       text.size = 4,
       text.col = "black")
dev.off()

cat("\n=== Exporting figures as PDF ===\n")

pdf("Figure_Calibration_Without_Arthralgia.pdf", width = 8, height = 7)
plot(cal_train_wo,
     xlim = c(0, 1), ylim = c(0, 1),
     xlab = "Predicted Probability",
     ylab = "Observed Probability",
     legend = FALSE,
     main = "Without Arthralgia (Training)",
     subtitles = FALSE,
     cex.main = 1.8, cex.lab = 1.6, cex.axis = 1.5)
abline(0, 1, col = "black", lty = 2, lwd = 3)
lines(cal_train_wo[, c("predy", "calibrated.orig")], type = "l", lwd = 3, col = "red")
lines(cal_train_wo[, c("predy", "calibrated.corrected")], type = "l", lwd = 3, col = "green")
legend(0.55, 0.35,
       c("Ideal", "Apparent", "Bias-corrected"),
```

```r
       lty = c(2, 1, 1),
       lwd = c(3, 3, 3),
       col = c("black", "red", "green"),
       bty = "n",
       cex = 1.5)
dev.off()

pdf("Figure_ROC_Without_Arthralgia.pdf", width = 8, height = 7)
plot(roc_wo, print.auc = TRUE, auc.polygon = TRUE, max.auc.polygon = TRUE,
     auc.polygon.col = "skyblue", print.thres = "best",
     main = "Without Arthralgia (Training)",
     col = "red", print.thres.col = "black",
     identity.col = "blue", identity.lty = 2,
     cex.main = 1.8, cex.lab = 1.6, cex.axis = 1.5)
dev.off()

pdf("Figure_DCA_Without_Arthralgia.pdf", width = 8, height = 7)
scidca(dca_glm_wo,
       threshold.line = TRUE,
       threshold.text = TRUE,
       text.size = 5,
       text.col = "black")
dev.off()

cat("\n=== Summary Table ===\n")

summary_wo <- data.frame(
  Metric = c("AUC", "AUC_CI_lower", "AUC_CI_upper",
             "Best_Threshold", "Sensitivity", "Specificity",
             "HL_Chi_square", "HL_DF", "HL_P_value"),
  Value = c(round(auc_wo, 3), round(ci_auc_wo[1], 3), round(ci_auc_wo[3], 3),
            round(best_threshold_wo, 3), round(best_sensitivity_wo, 3),
            round(best_specificity_wo, 3),
            if(exists("hl_test_wo")) round(hl_test_wo$statistic, 3) else NA,
```

```
        if(exists("hl_test_wo")) hl_test_wo$parameter else NA,
        if(exists("hl_test_wo")) round(hl_test_wo$p.value, 4) else NA)
)

print(summary_wo)
write.csv(summary_wo, "Model_Performance_Without_Arthralgia.csv", row.names =
FALSE)

cat("\n=== Analysis without Arthralgia completed ===\n")
cat("Files generated:\n")
cat("1. Multivariate_Analysis_Results_Without_Arthralgia.csv\n")
cat("2. HLtest_Result_Without_Arthralgia.csv\n")
cat("3. Calibration_Curve_Without_Arthralgia.png\n")
cat("4. ROC_Curve_Without_Arthralgia.png\n")
cat("5. DCA_Without_Arthralgia.png\n")
cat("6. Figure_Calibration_Without_Arthralgia.pdf\n")
cat("7. Figure_ROC_Without_Arthralgia.pdf\n")
cat("8. Figure_DCA_Without_Arthralgia.pdf\n")
cat("9. Model_Performance_Without_Arthralgia.csv\n")


# ============================================================
# E-value analysis for unmeasured confounding
# ============================================================

library(dplyr)

# ---------- 1. Read data ----------
df_train <- read.csv("Training dataset.csv")

# ---------- 2. Define predictors and their ORs/CIs ----------
vars <- c("Arthralgia", "Nail_lesions", "Uveitis", "Scalp_lesions", "Inverse_lesions")

or_values <- c(20.23, 5.98, 5.23, 2.97, 1.65)
```

```
ci_lower_values <- c(13.14, 3.69, 1.94, 1.45, 1.09)
ci_upper_values <- c(31.13, 9.68, 14.10, 6.10, 2.48)

# ---------- 3. Calculate P0 for each variable ----------
calc_p0 <- function(data, var) {
  unexposed <- data[data[[var]] == 0, ]
  return(sum(unexposed$PsA) / nrow(unexposed))
}

p0_values <- sapply(vars, function(v) calc_p0(df_train, v))

# ---------- 4. Define conversion functions ----------
or_to_rr <- function(or, p0) {
  return(or / ((1 - p0) + (p0 * or)))
}

calc_evalue <- function(rr) {
  if (is.na(rr) || rr <= 1) return(NA)
  return(rr + sqrt(rr * (rr - 1)))
}

# ---------- 5. Compute RR and E-value for each predictor ----------
results <- data.frame(
  Variable = vars,
  OR = or_values,
  CI_lower = ci_lower_values,
  CI_upper = ci_upper_values,
  P0_percent = round(p0_values * 100, 2),
  RR = NA,
  RR_lower = NA,
  RR_upper = NA,
  E_value = NA,
  E_value_lower = NA
)
```

```r
for (i in 1:nrow(results)) {
  p0 <- p0_values[i]

  results$RR[i] <- round(or_to_rr(results$OR[i], p0), 3)
  results$RR_lower[i] <- round(or_to_rr(results$CI_lower[i], p0), 3)
  results$RR_upper[i] <- round(or_to_rr(results$CI_upper[i], p0), 3)

  results$E_value[i] <- round(calc_evalue(results$RR[i]), 2)
  results$E_value_lower[i] <- round(calc_evalue(results$RR_lower[i]), 2)
}

# ---------- 6. Print results ----------
cat("========================================\n")
cat("Sample size:", nrow(df_train), "\n")
cat("PsA cases:", sum(df_train$PsA), "\n")
cat("PsA prevalence:", round(mean(df_train$PsA) * 100, 2), "%\n")
cat("========================================\n\n")

cat("=== P0 for each predictor ===\n")
for (i in 1:nrow(results)) {
  cat(results$Variable[i], ":", results$P0_percent[i], "%\n")
}

cat("\n=== E-value results ===\n")
print(results[, c("Variable", "OR", "RR", "E_value", "E_value_lower")])

# ---------- 7. Generate Supplementary Table ----------
cat("\n\n| Variable | OR (95% CI) | P₀ (%) | RR (95% CI) | E-value (RR) | E-value (RR lower) |\n")
cat("|----------|-------------|--------|-------------|--------------|---------------------|\n")
for (i in 1:nrow(results)) {
  cat("| ", results$Variable[i],
      " | ", results$OR[i], " (", results$CI_lower[i], "-", results$CI_upper[i], ")",
```

```
    " | ", results$P0_percent[i],
    " | ", results$RR[i], " (", results$RR_lower[i], "-", results$RR_upper[i], ")",
    " | ", results$E_value[i],
    " | ", results$E_value_lower[i],
    " |\n", sep = "")
}

# ---------- 8. Save results to CSV ----------
write.csv(results, "Evalue_results.csv", row.names = FALSE)
cat("\nResults saved to Evalue_results.csv\n")
```

Model Validation Code

```
setwd("C:/Users/GUO/Desktop")
library(readr)
library(pROC)
library(rms)
library(ResourceSelection)
library(ggplot2)
library(reshape2)
library(ggscidca)
```

```
# ========== 1. ==========
model_trained <- readRDS("training_model.rds")
cat("Loaded training model coefficients:\n")
print(coef(model_trained))

# ========== 2. ==========
valid <- read_csv("Validation dataset.csv", show_col_types = FALSE)
valid <- na.omit(valid)
valid <- as.data.frame(valid)

# ========== 3. ==========
valid$Arthralgia <- factor(valid$Arthralgia, levels = c(0, 1), labels = c("0", "1"))
valid$Nail_lesions <- factor(valid$Nail_lesions, levels = c(0, 1), labels = c("0", "1"))
valid$Scalp_lesions <- factor(valid$Scalp_lesions, levels = c(0, 1), labels = c("0", "1"))
valid$Inverse_lesions <- factor(valid$Inverse_lesions, levels = c(0, 1), labels = c("0",
"1"))
valid$Uveitis <- factor(valid$Uveitis, levels = c(0, 1), labels = c("0", "1"))
valid$Sex <- factor(valid$Sex, levels = c(0, 1), labels = c("0", "1"))
valid$Smoking_history <- factor(valid$Smoking_history, levels = c(0, 1), labels = c("0",
"1"))
valid$Family_psoriasis_history <- factor(valid$Family_psoriasis_history, levels = c(0,
1), labels = c("0", "1"))

valid$Educational_Level <- factor(valid$Educational_Level,
                                  levels = c(1, 2, 3),
                                  labels = c("1", "2", "3"),
                                  ordered = TRUE)

valid$PsA <- as.numeric(valid$PsA)

# ========== 4. ==========
continuous_vars <- c("Age", "Psoriasis_duration", "BSA", "BMI")
categorical_vars <- c("Sex", "Smoking_history", "Educational_Level",
                      "Family_psoriasis_history", "Uveitis", "Inverse_lesions",
```

```
                      "Scalp_lesions", "Nail_lesions", "Arthralgia")

PsA_0_desc <- c()
PsA_1_desc <- c()
p_values <- c()
test_methods <- c()

for (var in continuous_vars) {
  group0 <- valid[valid$PsA == 0, var]
  group1 <- valid[valid$PsA == 1, var]

  shapiro0 <- tryCatch(shapiro.test(group0)$p.value, error = function(e) 0)
  shapiro1 <- tryCatch(shapiro.test(group1)$p.value, error = function(e) 0)

  if (shapiro0 > 0.05 & shapiro1 > 0.05) {
    mean0 <- mean(group0, na.rm = TRUE)
    sd0 <- sd(group0, na.rm = TRUE)
    mean1 <- mean(group1, na.rm = TRUE)
    sd1 <- sd(group1, na.rm = TRUE)

    PsA_0_desc[var] <- paste0(round(mean0, 2), " ± ", round(sd0, 2))
    PsA_1_desc[var] <- paste0(round(mean1, 2), " ± ", round(sd1, 2))

    tt <- t.test(group0, group1)
    p_values[var] <- tt$p.value
    test_methods[var] <- "t-test"
  } else {
     med0 <- median(group0, na.rm = TRUE)
    min0 <- min(group0, na.rm = TRUE)
    max0 <- max(group0, na.rm = TRUE)
    med1 <- median(group1, na.rm = TRUE)
    min1 <- min(group1, na.rm = TRUE)
    max1 <- max(group1, na.rm = TRUE)
```

```
    PsA_0_desc[var] <- paste0(med0, " (", min0, ",", max0, ")")
    PsA_1_desc[var] <- paste0(med1, " (", min1, ",", max1, ")")

    wt <- wilcox.test(group0, group1)
    p_values[var] <- wt$p.value
    test_methods[var] <- "Mann-Whitney U"
  }
}

for (var in categorical_vars) {
  tab <- table(valid[, var], valid$PsA)

  if (nrow(tab) == 0) next

  total0 <- sum(tab[, "0"])
  total1 <- sum(tab[, "1"])

  desc0 <- c()
  desc1 <- c()

  for (i in 1:nrow(tab)) {
    level <- rownames(tab)[i]
    n0 <- tab[i, "0"]
    n1 <- tab[i, "1"]

    pct0 <- ifelse(total0 > 0, round(n0 / total0 * 100, 1), 0)
    pct1 <- ifelse(total1 > 0, round(n1 / total1 * 100, 1), 0)

    desc0 <- c(desc0, paste0(level, ": ", n0, " (", pct0, "%)"))
    desc1 <- c(desc1, paste0(level, ": ", n1, " (", pct1, "%)"))
  }
  PsA_0_desc[var] <- paste(desc0, collapse = "; ")
  PsA_1_desc[var] <- paste(desc1, collapse = "; ")
```

```
  if (any(dim(tab) == 1)) {
     p_values[var] <- NA
     test_methods[var] <- "N/A"
  } else {
    expected <- chisq.test(tab)$expected
    if (min(expected) < 5) {
      ft <- fisher.test(tab)
      p_values[var] <- ft$p.value
      test_methods[var] <- "Fisher's exact"
    } else {
      ct <- chisq.test(tab)
      p_values[var] <- ct$p.value
      test_methods[var] <- "Chi-square"
    }
  }
}

p_values_num <- as.numeric(p_values)

results_df <- data.frame(
  Variable = c(continuous_vars, categorical_vars),
  PsA_0 = PsA_0_desc,
  PsA_1 = PsA_1_desc,
  Test_method = test_methods,
  P_value = round(p_values_num, 4)
)

write.csv(results_df, "Descriptive_Statistics.csv", row.names = FALSE)
print(results_df)

# ========== 5.  ==========
valid$pred_prob <- predict(model_trained, newdata = valid, type = "response")

# ========== 6. ROC/AUC ==========
```

```
roc_valid <- roc(valid$PsA, valid$pred_prob, ci = TRUE)
auc_valid <- auc(roc_valid)
cat("AUC =", round(auc_valid, 3), "\n")
cat("95% CI:", round(ci.auc(roc_valid)[1], 3), "-", round(ci.auc(roc_valid)[3], 3), "\n")

youden_idx <- which.max(roc_valid$sensitivities + roc_valid$specificities - 1)
best_threshold <- roc_valid$thresholds[youden_idx]
best_sensitivity <- roc_valid$sensitivities[youden_idx]
best_specificity <- roc_valid$specificities[youden_idx]

cat("\n=== Optimal Threshold (Youden Index) ===\n")
cat("Best threshold:", round(best_threshold, 3), "\n")
cat("Sensitivity:", round(best_sensitivity, 3), "\n")
cat("Specificity:", round(best_specificity, 3), "\n")

png("ROC_Curve_Validation.png", width = 3500, height = 2800, res = 300)
plot(roc_valid, print.auc = TRUE, auc.polygon = TRUE,
     max.auc.polygon = TRUE, auc.polygon.col = "skyblue",
     print.thres = "best", main = "Validation dataset",
     col = "red", print.thres.col = "black",
     identity.col = "blue", identity.lty = 2)
dev.off()

# ========== 7. Calibration_Curve ==========
cal_data <- data.frame(PsA = valid$PsA, pred = valid$pred_prob)
dd <- datadist(cal_data)
options(datadist = "dd")

cal_model <- lrm(PsA ~ pred, data = cal_data, x = TRUE, y = TRUE)
cal_valid <- calibrate(cal_model, method = "boot", B = 1000, m = 50)

png("Calibration_Curve_Validation.png", width = 3500, height = 2800, res = 300)
plot(cal_valid, xlim = c(0, 1), xlab = "Predicted Probability",
     ylab = "Observed Probability", legend = FALSE,
```

```
  main = "Validation dataset", subtitles = FALSE)
abline(0, 1, col = "black", lty = 2, lwd = 2)
lines(cal_valid[, "predy"], cal_valid[, "calibrated.orig"], lwd = 2, col = "red")
lines(cal_valid[, "predy"], cal_valid[, "calibrated.corrected"], lwd = 2, col = "green")
legend(0.55, 0.35, legend = c("Ideal", "Apparent", "Bias-corrected"),
       lty = c(2, 1, 1), lwd = c(2, 2, 2), col = c("black", "red", "green"), bty = "n")
dev.off()

# ========== 8. Hosmer-Lemeshow  ==========
hl_valid <- hoslem.test(valid$PsA, valid$pred_prob, g = 10)
cat("Hosmer-Lemeshow χ² =", round(hl_valid$statistic, 3),
    ", p =", round(hl_valid$p.value, 3), "\n")

# ========== 9. DCA ==========
dca_data <- data.frame(PsA = valid$PsA, pred = valid$pred_prob)
dca_glm <- glm(PsA ~ pred, data = dca_data, family = binomial())

png("DCA_Validation.png", width = 3500, height = 2800, res = 300)
scidca(dca_glm,
       threshold.line = TRUE,
       threshold.text = TRUE,
       text.size = 4,
       text.col = "black")
dev.off()

# ========== 10. Clinical Impact Curve (CIC) ==========
library(rmda)
cic_data <- data.frame(PsA = valid$PsA, pred_prob = valid$pred_prob)
dca_fit <- decision_curve(PsA ~ pred_prob,
                          data = cic_data,
                          family = binomial(link = 'logit'),
                          thresholds = seq(0, 1, by = 0.01),
                          bootstraps = 50,
                          confidence.intervals = 0.95)
```

```
png("Clinical_Impact_Curve_Validation.png", width = 4000, height = 3000, res = 300)
plot_clinical_impact(dca_fit,
                     population.size = 1000,
                     cost.benefit.axis = TRUE,
                     n.cost.benefits = 8,
                     col = c('red', 'blue'),
                     confidence.intervals = TRUE,
                     ylim = c(0, 1000),
                     legend.position = "topright")

dev.off()

cat("Clinical Impact Curve saved to: Clinical_Impact_Curve_Validation.png\n")



optimal_threshold <- best_threshold
predicted_class <- ifelse(valid$pred_prob > optimal_threshold, 1, 0)
conf_mat <- table(Predicted = predicted_class, Actual = valid$PsA)
accuracy <- sum(diag(conf_mat)) / sum(conf_mat)

cat("Confusion Matrix:\n")
print(conf_mat)
cat("Accuracy =", round(accuracy * 100, 1), "%\n")

TP <- conf_mat[2,2]
TN <- conf_mat[1,1]
FP <- conf_mat[2,1]
FN <- conf_mat[1,2]

cat("Sensitivity =", round(TP/(TP+FN)*100, 1), "%\n")
cat("Specificity =", round(TN/(TN+FP)*100, 1), "%\n")

conf_df <- melt(conf_mat)
```

```
colnames(conf_df) <- c("Prediction", "Reference", "Count")
conf_df$Prediction <- factor(conf_df$Prediction, levels = c(0, 1), labels = c("No PsA",
"PsA"))
conf_df$Reference <- factor(conf_df$Reference, levels = c(0, 1), labels = c("No PsA",
"PsA"))

png("Confusion_Matrix_Validation.png", width = 3500, height = 3000, res = 300)
ggplot(conf_df, aes(x = Reference, y = Prediction, fill = Count)) +
  geom_tile(color = "white", linewidth = 1) +
  geom_text(aes(label = Count), color = "white", size = 6, fontface = "bold") +
  scale_fill_gradient(low = "steelblue", high = "darkred", name = "Count") +
  theme_minimal(base_size = 14) +
  labs(title = "Confusion Matrix (External Validation, Threshold = 0.384)",
       x = "Actual", y = "Predicted") +
  theme(axis.text = element_text(size = 12, color = "black"),
        axis.title = element_text(size = 14, face = "bold"),
        plot.title = element_text(size = 16, face = "bold", hjust = 0.5),
        panel.grid = element_blank())
dev.off()

mae_value <- mean(abs(valid$pred_prob - valid$PsA))

valid_summary <- data.frame(
  Metric = c("AUC", "AUC 95% CI", "Accuracy", "Sensitivity", "Specificity",
             "Hosmer-Lemeshow χ²", "Hosmer-Lemeshow P", "MAE"),
  Value = c(
    round(auc_valid, 3),
    paste(round(ci.auc(roc_valid)[1], 3), "-", round(ci.auc(roc_valid)[3], 3)),
    paste0(round(accuracy * 100, 1), "%"),
    paste0(round(TP/(TP+FN)*100, 1), "%"),
    paste0(round(TN/(TN+FP)*100, 1), "%"),
    round(hl_valid$statistic, 3),
    round(hl_valid$p.value, 4),
    round(mae_value, 4)  )
```

```
)

cat("\n=== Validation Summary ===\n")
print(valid_summary)
write.csv(valid_summary, "Validation_Summary.csv", row.names = FALSE)

cat("\n=== External Validation Completed ===\n")

# ============================================================
# Export all validation figures as PDF (vector format)
# ============================================================

# Figure 1: ROC Curve (Validation)
pdf("Figure_ROC_Validation.pdf", width = 8, height = 7)
plot(roc_valid,
    print.auc = TRUE,
    auc.polygon = TRUE,
    max.auc.polygon = TRUE,
    auc.polygon.col = "skyblue",
    print.thres = "best",
    main = "Validation dataset",
    col = "red",
    lwd = 3,
    cex.main = 1.8,
    cex.lab = 1.6,
    cex.axis = 1.5)
dev.off()

# Figure 2: Calibration Curve (Validation)
pdf("Figure_Calibration_Validation.pdf", width = 8, height = 7)
plot(cal_valid,
    xlim = c(0, 1),
    ylim = c(0, 1),
    xlab = "Predicted Probability",
```

```r
    ylab = "Observed Probability",
    legend = FALSE,
    main = "Validation dataset",
    subtitles = FALSE,
    cex.main = 1.8,
    cex.lab = 1.6,
    cex.axis = 1.5)
abline(0, 1, col = "black", lty = 2, lwd = 3)
lines(cal_valid[, "predy"], cal_valid[, "calibrated.orig"], lwd = 3, col = "red")
lines(cal_valid[, "predy"], cal_valid[, "calibrated.corrected"], lwd = 3, col = "green")
legend(0.55, 0.35,
       legend = c("Ideal", "Apparent", "Bias-corrected"),
       lty = c(2, 1, 1),
       lwd = c(3, 3, 3),
       col = c("black", "red", "green"),
       bty = "n",
       cex = 1.5)
dev.off()

# Figure 3: DCA (Validation)
pdf("Figure_DCA_Validation.pdf", width = 8, height = 7)
scidca(dca_glm,
       threshold.line = TRUE,
       threshold.text = TRUE,
       text.size = 5,
       text.col = "black")
dev.off()

# Figure 4: Clinical Impact Curve (Validation)
pdf("Figure_CIC_Validation.pdf", width = 10, height = 8)
plot_clinical_impact(dca_fit,
                     population.size = 1000,
                     cost.benefit.axis = TRUE,
                     n.cost.benefits = 8,
```

```
                    col = c('red', 'blue'),
                    confidence.intervals = TRUE,
                    ylim = c(0, 1000),
                    legend.position = "topright",
                    cex.axis = 1.5,
                    cex.lab = 1.6,
                    cex.main = 1.8)
dev.off()

# Figure 5: Confusion Matrix (Validation)
pdf("Figure_ConfusionMatrix_Validation.pdf", width = 8, height = 7)
ggplot(conf_df, aes(x = Reference, y = Prediction, fill = Count)) +
  geom_tile(color = "white", linewidth = 1) +
  geom_text(aes(label = Count), color = "white", size = 8, fontface = "bold") +
  scale_fill_gradient(low = "steelblue", high = "darkred", name = "Count") +
  theme_minimal(base_size = 16) +
  labs(title = "Confusion Matrix (External Validation)",
       x = "Actual", y = "Predicted") +
  theme(axis.text = element_text(size = 14, color = "black"),
        axis.title = element_text(size = 16, face = "bold"),
        plot.title = element_text(size = 18, face = "bold", hjust = 0.5),
        panel.grid = element_blank())
dev.off()

cat("\n=== All validation figures exported as PDF ===\n")
cat("Figure_ROC_Validation.pdf\n")
cat("Figure_Calibration_Validation.pdf\n")
cat("Figure_DCA_Validation.pdf\n")
cat("Figure_CIC_Validation.pdf\n")
cat("Figure_ConfusionMatrix_Validation.pdf\n")
```
