# Supplementary material for: A Clinically Accessible Nomogram for Identifying Likely Psoriatic Arthritis Among Patients with Psoriasis: A Multicenter Cross-Sectional Study
Source: J Clin Med. 2026 Aug 11;15(16):6215. doi: 10.3390/jcm15166215 (PMC13512928; doi:10.3390/jcm15166215)
Supplement: Supplementary file 1 [file jcm-15-06215-s001.zip › Table S1.pdf]

Table S1. E-value analysis for unmeasured confounding

Variables	OR (95% CI)	P_0 (%)	RR (95% CI)	E-value (RR)	E-value (RR lower)
Uveitis	5.23 (1.94–14.10)	23.45	2.63 (1.59–3.46)	4.69	2.56
Inverse lesions	1.65 (1.09–2.48)	18.75	1.47 (1.07–1.94)	2.30	1.35
Scalp lesion	2.97 (1.45–6.10)	12.03	2.40 (1.38–3.78)	4.24	2.10
Nail lesion	5.98 (3.69–9.68)	8.44	4.21 (3.01–5.59)	7.89	5.46
Arthralgia	20.23 (13.14–31.13)	9.97	6.94 (5.95–7.78)	13.35	11.37

RRs were derived from ORs using the formula $RR = OR / [(1 - P_0) + (P_0 \times OR)]$, where P_0 is the specific PsA prevalence in the unexposed group for each predictor.
