# Supplementary material for: A Clinically Accessible Nomogram for Identifying Likely Psoriatic Arthritis Among Patients with Psoriasis: A Multicenter Cross-Sectional Study
Source: J Clin Med. 2026 Aug 11;15(16):6215. doi: 10.3390/jcm15166215 (PMC13512928; doi:10.3390/jcm15166215)
Supplement: Supplementary file 1 [file jcm-15-06215-s001.zip › Table S2.pdf]

Table S2. Multivariable logistic regression analysis of the model excluding arthralgia

Variables	B	SE	OR	95%CI	Z	P
Intercept	-3.298	0.328	0.04	0.02-0.07	-10.055	＜0.001
Uveitis	1.868	0.435	6.48	2.76-15.2	4.298	＜0.001
Inverse lesions	0.605	0.171	1.83	1.31-2.56	3.532	＜0.001
Scalp lesion	0.679	0.298	1.97	1.1-3.54	2.276	0.0228
Nail lesion	1.849	0.208	6.35	4.23-9.55	8.9	＜0.001
